# Association of Epistatic Effects of lncRNA *GAS5*, *miR*-*146a*, *IRAK-1*, and *miR-155* Genetic Variants with Multiple Sclerosis Risk and Severity

**DOI:** 10.1007/s12035-025-04876-8

**Published:** 2025-04-15

**Authors:** Ghada Ayeldeen, Olfat G. Shaker, Mohammed Gomaa, Mostafa M. Magdy, Nourhan Elsamaloty, Ahmed S. Kamel, Mahmoud A. Senousy

**Affiliations:** 1 Department of Medical Biochemistry and Molecular Biology, Faculty of Medicine, Cairo University, Cairo, Egypt; 2 Department of Neurology, Faculty of Medicine, Fayoum University, Fayoum, Egypt; 3 Department of Biochemistry, Faculty of Pharmacy and Drug Technology, Egyptian Chinese University, Cairo, 11786 Egypt; 4 Department of Pharmacology and Toxicology, Faculty of Pharmacy, Cairo University, Cairo, 11562 Egypt; 5 Department of Pharmacology and Toxicology, Faculty of Pharmacy and Drug Technology, Egyptian Chinese University, Gesr El Suez St, Cairo, PO 11786 Egypt; 6 Department of Biochemistry, Faculty of Pharmacy, Cairo University, Cairo, 11562 Egypt

**Keywords:** Epistasis, LncRNA, MiRNA, RRMS, SNP, SPMS

## Abstract

The complex genetic architecture of heritability in multiple sclerosis (MS) remains undisclosed mainly. Epistasis (gene–gene interaction) substantially impacts MS; however, it is largely unexplored, especially among the non-coding RNA genes and their targets. The long non-coding RNA *GAS5* exacerbates demyelination and sponges *miR*-*146a* and *miR-155*, impeccable contributors to MS pathogenesis. *miR*-*146a* negatively regulates the immune responses by targeting *IRAK-1*. We investigated the association of epistatic effects and haplotypes of five single nucleotide polymorphisms (SNPs), *GAS5* rs2067079, *miR*-*146a* rs2910164 and rs57095329, *IRAK-1* rs3027898, and *miR-155* rs767649, with the risk of MS and its phenotypes. The expression quantitative trait locus (eQTL) associated with these variants was explored through bioinformatics analysis. The study enrolled 116 MS patients and 120 healthy controls. No strong linkage disequilibrium (*D*′ ≥ 0.8) was detected among the studied SNPs. SNP-SNP interactions overlaid an overall magnified risk of MS and its phenotypes compared to the single-locus effects. After adjustment for multiple comparisons, the most significant interactions associated with the risk of overall MS and secondary-progressive MS were rs2067079-rs2910164, rs2910164-rs57095329, and rs3027898-rs767649. The last two former SNP-SNP interactions were highly associated with relapsing–remitting MS risk. The same pattern of interactions, as observed in association with MS risk, was female-specific. The CCAAA haplotype (alleles in the order of rs2067079, rs2910164, rs57095329, rs3027898, and rs767649) was protective against MS risk (CCAAA vs. CGAAT, adjusted OR = 0.14, 95% CI = 0.03–0.69, *P* = 0.009). Among MS patients, harboring the CGACT and CGAAT haplotypes was more prevalent in females and males, respectively. MS patients having EDSS ≥ 6 had a significantly higher frequency of the CCGCA haplotype than those with EDSS < 6. Functional analysis revealed rs2067079, rs57095329, and rs767649 as strong cis-eQTL regulating multiple genes, particularly in the brain and immune system. We propose that a magnified combined effect of *GAS5*, *miR*-*146a*, *IRAK-1*, and *miR*-*155* genetic variants via epistatic interactions might impact the risk of MS and its phenotypes and could help in the risk stratification of MS patients.

**Supplementary Information:**

The online version contains supplementary material available at 10.1007/s12035-025-04876-8.

## Introduction

Multiple sclerosis (MS) is a long-lasting autoimmune neurodegenerative disease characterized by the main triad symptoms of chronic inflammation, demyelination, and gliosis of the brain and spinal cord [1]. These symptoms are instigated by autoreactive CD4 + T cells, the main driver of MS pathogenesis. CD4 + T cells are activated in the peripheral blood, cross the blood–brain barrier along with activated myeloid cells and B lymphocytes, and then reactivated and spread in the central nervous system (CNS), creating an inflammatory environment. This process culminates in axon demyelination and degeneration, microglial activation, and neuronal death, which are the hallmarks of MS. These structural abnormalities result in dysfunctional neurological episodes and disability [2].

More than two million people are affected by MS worldwide, with over 75% of them being women. Currently, MS cases are primarily classified into four different clinical phenotypes: clinically isolated syndrome (CIS), relapsing–remitting MS (RRMS), primary-progressive MS (PPMS), and secondary-progressive MS (SPMS) [3]. RRMS is the most common clinical type (~ 85%), with about 15 to 30% of RRMS patients progressing over time to SPMS [4, 5].

Compelling evidence suggests that an interaction occurs between genetic, epigenetic, environmental, and lifestyle factors, which could influence the susceptibility to MS [6]. Various population, familial, and molecular studies have documented that the vulnerability to MS is significantly impacted by genetic variations located within or close to immune-related and CNS-related genes [7–10]. These studies provided evidence that MS has a polygenic pattern of inheritance [7–11]. Over 13 genome-wide association studies (GWAS) have been conducted on MS patients and control subjects, accounting for about 48% of MS heritability [10, 12]. These GWAS have identified 32 major histocompatibility complex (MHC) variants and over 200 non-MHC candidate variants associated with MS, with the haplotype HLA-DRB1*15:01 (MHC, chromosome 6p21.3) having the most substantial impact [10, 12]. However, a large part of the complex genetic architecture of heritability in MS is still undisclosed.

Interestingly, genetic studies have found no single mutation that contributes to a large effect on MS susceptibility, and the effect of individual variants of susceptibility genes remains modest [10]. Indeed, these variants converge into pathways related to T cells and adaptive immune response [10–13]; thereby, it is plausible that multiple gene–gene interactions (epistasis) and/or gene-environment interactions could magnify the overall genetic risk for MS [11]. In addition, the causal variants within risk loci are often questioned, and there are challenges in their interpretation [10, 13]. In particular, genetic variants in long non-coding RNAs (lncRNAs) and microRNAs (miRNAs) are not often highlighted in interpretation strategies.

lncRNAs and miRNAs are deregulated in different immune cells, circulation, and cerebrospinal fluid of MS patients and in experimental autoimmune encephalomyelitis (EAE) animal models of MS [14–16]. Mainly, lncRNAs act as miRNA sponges, and both are entangled in immune regulation, myelination, and remyelination processes, as well as the onset and progression of MS [14]. The lncRNA growth-arrest specific transcript 5 (*GAS5*) is of interest, as it suppresses the human microglial M2 polarization and exacerbates demyelination [17]. Intriguingly, *GAS5* sponges *miR*-*146a* and *miR-155* in various inflammatory conditions, including neuroinflammation [18–21]. In particular, *miR*-*146a* and *miR-155* are impeccable contributors to MS pathogenesis in humans [22–24] and the EAE model [25, 26]. Indeed, *miR*-*146a* negatively regulates the immune and inflammatory responses by targeting the tumor necrosis factor (TNF) receptor-associated family (TRAF)−6 and interleukin (IL)−1 receptor-associated kinase-1 (*IRAK-1*) to suppress nuclear factor-κB (NF-κB) activation and subsequent cytokine production [27]. Furthermore, *miR-155* is a principal regulator of innate and adaptive immune responses [28], and its silencing attenuates demyelination in EAE [25, 26].

Interestingly, genetic variants in *GAS5* (rs2067079), *miR*-*146a* (rs2910164 and rs57095329), *IRAK-1* (rs3027898), and *miR-155* (rs767649) have been connected to MS predisposition in modestly sized Egyptian studies [29–31]. Extensive-sized studies in Iranian and Russian cohorts uncovered the association of *GAS5* rs2067079 [32] and *miR*-*146a* rs2910164 [33, 34] with MS risk. In addition, an Italian study unveiled the association of the *miR-155* genetic locus, nearby rs767649, with MS risk [35]. However, these studies have focused on studying the single-locus effects of these variants, leaving a gap in knowledge about the impact of their epistatic effects on MS risk. Epistasis has been disclosed to largely influence the risk of MS and might account for part of its unexplained genetic heritability [11]. Nevertheless, only a few researchers have studied the influence of epistatic effects of SNPs on MS heritability [36–38]. Thereby, appraising the association of epistatic effects of potential susceptibility genes can offer new opportunities for understanding the complex genetic variance of MS.

This study is aimed at identifying the epistatic effects and haplotypes of *GAS5*, *miR*-*146a*, its target *IRAK-1*, and *miR-155* SNPs concerning the susceptibility to MS and its subtypes. In addition, we evaluated the association of these interactions in risk-stratified groups. The association of haplotypes of these SNPs with the patient’s demographic and clinical data was explored. Moreover, functional analysis was conducted to visualize the expression quantitative trait locus (eQTL) associated with the studied variants.

## Subjects and Methods

### Subjects

This case–control study encompassed an overall sample of 236 unrelated ethnic Egyptian patients divided into 116 patients clinically confirmed with MS and 120 age- and sex-matched healthy controls. To ensure a relatively homogenous ethnicity of the sampled population, our study was conducted exclusively on a population sample from Fayoum Governorate. MS patients were registered as inpatients or outpatients in the Department of Neurology, Faculty of Medicine, Fayoum University, Fayoum, Egypt. The demographic data (age, age at disease onset, and gender), medical histories, and the results of clinical examinations, including the expanded disability status scale (EDSS) and MS type, were recorded for every participant. As MS primarily manifests in young adults (20–40 years) [39], the age at onset level (the age at which MS symptoms first appeared in the study participants) was set at 30 years, which matches the mean age at onset in the current study.

The revised McDonald’s criteria 2017 were set as the guideline for MS diagnosis, applying clinical and MRI-based evidence to improve diagnostic accuracy [40]. MS patients were classified into two subtypes: eighty-four patients suffer from RRMS and 32 from SPMS. The degree of neurological disability of MS patients over time was evaluated using the EDSS [41]. An EDSS score ≥ 6 was set as the threshold of severe MS. This cut-off value indicates a significant disability, with patients experiencing loss of independent ambulation, requiring assistance such as a cane or other walking aid to walk approximately 100 m [42]. According to their EDSS, MS patients were divided into two subgroups: EDSS < 6 representing mild/moderate MS and EDSS ≥ 6 representing severe MS.

The primary criteria for inclusion were adult patients (18 years or higher) of both genders with a clinical diagnosis of MS. Exclusion criteria were patients having other autoimmune or inflammatory, chronic infectious or oncologic diseases, pregnancy, or patients suspected to be on drug or alcohol abuse. MS patients with active disease, including those experiencing current relapses, were excluded. This was confirmed using MRI with contrast, ensuring that only patients in a stable disease state were included in the analysis.

The data of the enrolled control subjects showed no signs of neurological disorders. Their medical history and clinical status were free from any neurological or autoimmune disease, and they had no family history of MS.

### SNPs Selection

To be relevant to the interaction analysis, the SNPs’ selection was based on three criteria: a global minor allele frequency (MAF) > 0.1, a previously reported association with MS, and a reported functional relation with their transcript expression. Selecting the MAF threshold depends on the sample size. Given the modest sample size of our study, we set a higher MAF threshold (> 0.1) to reduce the risk of false positives, increase the likelihood of replication, and maintain sufficient statistical power for detecting meaningful associations. The position, MAF, and function of the studied SNPs (*GAS5* rs2067079, *miR*-*146a* rs2910164, rs57095329, *IRAK-1* rs3027898, and *miR-155* rs767649) are listed in Table 1. The global MAF of these SNPs is > 0.1, and they have been reported to be functional, altering the expression, secondary structure, and/or function of their transcripts [43–49].

**Table 1:** SNPs position, MAF, and function

Gene	SNP, major/minor allele	Chromosomal location	Global MAF^#^	Highest population MAF^#^	Study MAF (in the control group)	Function
*GAS5*	rs2067079 (C/T)	1:173866073	*T* = 0.19	0.39	0.27	Non-coding transcript exon variant
*miR-146a*	rs2910164 (C/G)	5:160485411	*C* = 0.29	0.4	0.45	Mature miRNA variant
	rs57095329 (A/G)	5:160467840	*G* = 0.14	0.24	0.46	Intron variant, located at promoter region
*IRAK-1*	rs3027898 (A/C)	X:154010439	*C* = 0.25	0.48	0.2	Regulatory region variant
*miR-155*	rs767649 (A/T)	21:25572410	*T* = 0.15	0.44	0.35	Intron variant

*MAF*, minor allele frequency. ^#^According to ensembl online database (Human CRCh38.p14, release 112, May 2024)

Out of four reported SNPs in the *GAS5* gene (rs2067079, rs6790, rs145204276, and rs55829688), the rs2067079 was the only fitting. According to the GWAS catalog (https://www.ebi.ac.uk/gwas/), the rs2067079 SNP is a GWAS SNP (*P* = 4 × 10^−16^). Additionally, this SNP has been previously associated with MS in population studies [29, 32]. Furthermore, rs2067079 is located in an active promoter or strong enhancer region of the *GAS5* gene, with a reported influence on the transcript secondary structure, stability, and function [50].

Three functional variants of *miR*-*146a* (rs2910164, rs57095329, and rs2431697) were reported in association with certain autoimmune diseases in a meta-analysis [51]. Only rs2910164 and the promoter SNP rs57095329 were associated with MS risk [30, 31, 33]. These common polymorphic sites of *miR*-*146a* were evidenced to alter the function and expression of *miR*-*146a* [44–46]. The *IRAK-1* gene has four genetic loci (rs1059702, rs7061789, rs1059703, and rs3027898) on the X chromosome. These loci have been associated with the risk of several autoimmune diseases, especially in females, and their haplotype was shown to elevate *IRAK-1* expression [47]. However, only rs3027898 was associated with MS in a previous Egyptian study [30] and is commonly reported in association with autoimmune diseases in independent cohorts [52, 53], especially across the Egyptian population [30, 54]. Among the four identified loci in the *miR-155* gene, rs767649 is the most studied, and a previous association of this variant with MS risk was documented [31]. This variant, located in the flanking regulatory region of the *miR-155* gene, is functional and could increase the expression of miR-155 [48, 49].

### DNA Genotyping

At their enrollment, whole blood samples (5 to 6 mL) were collected from all participants on EDTA vacutainer tubes. Genomic DNA was extracted from blood using the QIAamp DNA MiniKit (Qiagen). The yield and purity of DNA were measured using the NanoDrop 2000 (Thermo Fisher Scientific). The TaqMan allelic discrimination assay (Applied Biosystems; Thermo Fisher Scientific, Inc.) was employed for genotyping using primer/probe sets for *GAS5* rs2067079 C/T (assay ID: C__16166809_20), *miR*-*146a* rs2910164 C/G (C_15946974_10), *miR*-*146a* rs57095329 A/G (C_90078480_10), *IRAK-1* rs3027898 A/C (C_15765198_10), and *miR-155* rs767649 A/T (C_2212229_10) (Thermo Fisher Scientific, Inc.) on the Rotor Gene Q real-time PCR system (Qiagen, Inc.).

For quality control, we used negative controls with no template to ensure no contamination and check the specificity of PCR. Moreover, every PCR run included both patient and control samples. Additionally, a subset of randomly selected samples (~ 10% of the total) was re-genotyped in duplicate, and the concordance rate was > 99%.

The present study analyzed SNPs in different genetic models, as previously described [55], to confirm the association between the investigated genes and MS risk. In brief, the additive (codominant) and log-additive (multiplicative) models assume that the major allele homozygote (wild-type, MM), the heterozygote (Mm), and the minor allele homozygote (mutant, mm) are associated with the lowest, the intermediate, and the highest risk, respectively (or the reverse). In these models, MM is set as a reference to investigate the prevalence of Mm and mm. In the dominant model, MM is set as a reference to evaluate the prevalence of minor allele-associated genotypes (Mm + mm). The recessive model compares the minor homozygous genotype (mm) against the major allele-associated genotypes (MM + Mm). The overdominant model assumes the heterozygote has the strongest impact and investigates its prevalence against the MM + mm genotypes. These models illustrate a subject-level phenomenon, whereas the allelic model investigates the impact of individual alleles on the disease.

### Functional Expression Quantitative Trait Locus (eQTL) Analysis

For analysis of the regulatory functions of the tested variants, we employed the FIVEx browser (https://fivex.sph.umich.edu/) for analyzing the cis-eQTLs associated with these variants and whether they are tissue-specific. The FIVEx is an interactive eQTL browser across public datasets hosting 16 studies from the EPI eQTL catalog [56]. In addition, the eQTLGen phase I (https://www.eqtlgen.org/trans-eqtls.html) was employed to check both the cis- and trans-eQTL effects. Trans-eQTL effects were analyzed using the ncRNA-eQTL database (http://ibi.hzau.edu.cn/ncRNA-eQTL/trans.php). Single-tissue eQTLs were confirmed using the Genotype-Tissue Expression (GTEx) portal (GTEx Analysis Release V10) (https://www.gtexportal.org/home/).

### Statistical Analysis

All association, interactions, haplotype, and linkage disequilibrium (LD) analyses were conducted using the SNPStats online software. Logistic regression models were constructed using age and sex as confounding factors. The adjusted odds ratio (OR) with a 95% confidence interval (CI) was used to express the associations. In the single-locus analysis, the major allele or the major homozygous genotype in the control group was set as a reference. In the haplotype analysis, the reference haplotype was the haplotype having the highest total (pooled) frequency from cases and controls (CGAAT, alleles in the order of rs2067079, rs2910164, rs57095329, rs3027898, and rs767649). The demographic and clinical data were statistically analyzed employing the GraphPad Prism software version 6.0 (GraphPad Software, CA) and are expressed as mean ± standard deviation, median (IQR), or number (percentage) when appropriate. When proper, the t-test, or Fisher exact test, was conducted to compare data from two independent groups. The statistical significance level was set as a two-tailed *P*-value < 0.05.

The codominant model was used to examine the SNP-SNP interactions, and the Bonferroni correction was employed to account for multiple comparisons. A significance threshold of Bonferroni *P* < 0.01 [0.05/5 independent tests (SNPs genotyped)] was set to make sure that type I errors were strictly controlled in the SNP-SNP interaction analysis. For the single-locus analysis in different genetic models, Bonferroni *P* < 0.01 threshold was used (0.05/5 subject-level genetic models tested).

The sample size was estimated using the web-based Power Calculator for Genetic Studies (https://csg.sph.umich.edu/abecasis/cats/gas_power_calculator/) with a power of 0.81. The following assumptions were considered: case/control ratio 1:1, a multiplicative disease model, a level of significance α = 0.05, a predicted risk allele frequency of ≥ 0.25, and a genotype relative risk of ≥ 1.75, as previously described [29, 57], using a prevalence of MS in Egypt of 14.1/100,000 as reported in prior epidemiologic studies [58, 59].

## Results

### Demographic and Clinical Data of MS Patients and Healthy Controls

Table 2 shows the demographic and clinical characteristics of the studied groups. The MS group showed female predominance with a female: male ratio = 2.7:1. Most patients presented with RRMS, while 27.6% (just above the 25% percentile) presented with SPMS. The EDSS was variable, with 23.3% (below the 25% percentile) of patients having severe MS (EDSS ≥ 6).

**Table 2:** Demographic and clinical data of studied groups

Parameters	Control (*n* = 120)	MS (*n* = 116)	*P-*value
Age (years)	33.82 ± 9.87	31.58 ± 8.68	0.07
Range	18–54	19–56	
< 30	43 (35.8)	56 (48.3)	
≥ 30	77 (64.2)	60 (51.7)	
Age of onset	––	31.37 ± 8.95	
Sex, *n* (%)			0.39
Male	40 (33.3)	32 (27.6)	
Female	80 (66.7)	84 (72.4)	
EDSS, *n* (%)			
< 6	––	89 (76.7)	
≥ 6	––-	27 (23.3)	
Median (IQR)	––	3.5 (2 – 5.5)	
MS type, *n* (%)			
RRMS	––	84 (72.4)	
SPMS	––	32 (27.6)	

Values are presented as mean ± SD, median (IQR), or number (percentage). The t-test, or Fisher exact test, was conducted. Statistical significance was set at *P* < 0.05

### Association of Single-Locus Effects with the Risk of MS and Its Subtypes

The five studied SNPs have MAF > 0.1 in the control group (Table 1). MAFs of *GAS5* rs2067079 (C/T) and *IRAK-1* rs3027898 (A/C) were close to the global MAF, while those for *miR*-*146a* rs2910164 (C/G) and *miR-155* rs767649 (A/T) were close to the highest population MAF reported in the ensemble online database (Human CRCh38.p14, release 112, May 2024). The *miR*-*146a* rs57095329 (A/G) MAF was higher than the highest population MAF. For all studied SNPs, the distribution of genotypes in the control group did not deviate from the Hardy–Weinberg equilibrium (*P* > 0.05), indicating their validity for analysis (Supplementary Table S1).

Table 3 shows the single-locus effects of the five tested SNPs on the risk of MS and its subtypes, RRMS and SPMS. Compared to healthy controls, our results demonstrated that the minor TT genotype (recessive model), minor C, and T alleles (allelic model) of *GAS5* rs2067079, *IRAK-1* rs3027898, and *miR-155* rs767649 SNPs, respectively, were candidate risk factors for developing MS. In contrast, the minor C and G alleles of *miR*-*146a* rs2910164 and rs57095329, respectively, were protective (allelic model) (*P* < 0.05, before Bonferroni correction). By sorting ORs (Table 3), the *miR*-*146a* rs57095329 minor G allele, with the smallest OR among the studied SNPs, showed the strongest (protective) impact (G vs. A, adjusted OR (95% CI) = 0.19 (0.12–0.32), *P* < 0.0001), after adjusting with age and sex as confounders. In other words, the major A risk allele of *miR*-*146a* rs57095329, with the highest OR, conferred the highest risk (5.26-fold) for MS.

**Table 3:** Association of studied SNPs with the risk of MS and its subtypes

SNP	Allele/genotype	Control (*n* = 120)	MS (*n* = 116)	Adjusted OR (95% CI), *P*^*a*^-value	RRMS (*n* = 84)	Adjusted OR (95% CI), *P*^*a*^-value	SPMS (*n* = 32)	Adjusted OR (95% CI), *P*^*a*^-value
*GAS5* rs2067079 (C/T)	CC + CT^$^	116 (96.7%)	100 (86.2%)	1.00	70 (83.3%)	1.00	30 (93.8%)	1.00
	TT	4 (3.3%)	16 (13.8%)	**4.62 (1.48–14.43), 0.0035**^**#**^	14 (16.7%)	**5.78 (1.81–18.54), 0.0011**^**#**^	2 (6.2%)	1.77 (0.30–10.63), 0.54
*miR-146a* rs2910164 (C/G)	G	132 (0.55)	144 (0.62)	1.00	109 (0.65)	1.00	35 (0.55)	1.00
	C	108 (0.45)	88 (0.38)	**0.62 (0.40–0.94), 0.022**	59 (0.35)	**0.51 (0.31–0.82), 0.004**^**#**^	29 (0.45)	0.94 (0.50–1.75), 0.84
*miR-146a* rs57095329 (A/G)	A	130 (0.54)	193 (0.83)	1.00	147 (0.88)	1.00	46 (0.72)	1.00
	G	110 (0.46)	39 (0.17)	**0.19 (0.12–0.32), < 0.0001**^**#**^	21 (0.12)	**0.13 (0.07–0.24), < 0.0001**^**#**^	18 (0.28)	**0.44 (0.23–0.86), 0.01**
*IRAK-1* rs3027898 (A/C)	A	192 (0.8)	138 (0.59)	1.00	110 (0.65)	1.00	28 (0.44)	1.00
	C	48 (0.2)	94 (0.41)	**2.25 (1.50–3.35), < 0.0001**^**#**^	58 (0.35)	**1.80 (1.16–2.79), 0.0085**^**#**^	36 (0.56)	**4.19 (2.28–7.70), < 0.0001**^**#**^
*miR-155* rs767649 (A/T)	A	157 (0.65)	91 (0.39)	1.00	58 (0.35)	1.00	33 (0.52)	1.00
	T	83 (0.35)	141 (0.61)	**3.64 (2.28–5.79), < 0.0001**^**#**^	110 (0.65)	**4.79 (2.78–8.27), < 0.0001**^**#**^	31 (0.48)	1.86 (0.99–3.48), 0.051

The association analysis was performed using SNPStats online software. The allelic model is presented. When the allelic model is non-significant, the best fit model is presented. ^$^Represents the recessive model (the best fit model of the *GAS5* SNP based on the lowest Akaike Information Criterion and Bayesian Information Criterion). Bold indicates statistical significance, *P* < 0.05. Data are presented in the sequence of OR (95% Cl), *P*-value. ^a^Adjusted for age and sex in a logistic regression model. ^#^Significant after adjustment for multiple comparisons (Bonferroni *P* < 0.01 threshold)

The same pattern of risk genotypes/alleles of these SNPs significantly applied for RRMS compared with the healthy control group (Table 3). By sorting ORs among the studied SNPs (Table 3), the *miR*-*146a* rs57095329 minor G allele (having the lowest OR) showed the strongest impact (G vs. A, adjusted OR (95% CI) = 0.13 (0.07–0.24), *P* < 0.0001), whereas the major A risk allele (having the highest OR) was associated with the highest risk for RRMS (A vs. G, adjusted OR (95% CI) = 7.69 (4.16–14.28), *P* < 0.0001), after adjusting with age and sex as confounding factors.

For SPMS, only *miR*-*146a* rs57095329 and *IRAK-1* rs3027898 posed a significant association with its risk in the allelic model in comparison with the healthy controls (*P* = 0.01, *P* < 0.0001, respectively) (Table 3). In a separate analysis, the *miR-155* rs767649 SNP was associated with an elevated risk of SPMS in the codominant model (TT vs. AA, adjusted OR (95% CI) = 3.7 (1.07–12.90), *P* = 0.032); however, it did not survive the Bonferroni correction. Among these, *IRAK-1* rs3027898 minor C risk allele conferred the strongest impact on SPMS risk (C vs. A, adjusted OR (95% CI) = 4.19 (2.28–7.70), *P* < 0.0001).

The genotype distribution of the studied SNPs in the control and MS groups is detailed in Supplementary Tables S2 to S6. After adjusting for age and sex, these SNPs were associated with the risk of MS in at least one genetic model. When a strict *P*-value threshold (Bonferroni *P* < 0.01) was applied to correct for multiple testing, four of the five SNPs remained associated with MS risk in at least one genetic model, while *miR*-*146a* rs2910164 lost statistical significance. Notably, the association pattern of SNPs with the risk of RRMS (5 SNPs) and SPMS (rs57095329 and rs3027898) remained significant when Bonferroni *P* < 0.01 was applied (Table 3).

### Results of LD Analysis

**Fig. 1:**
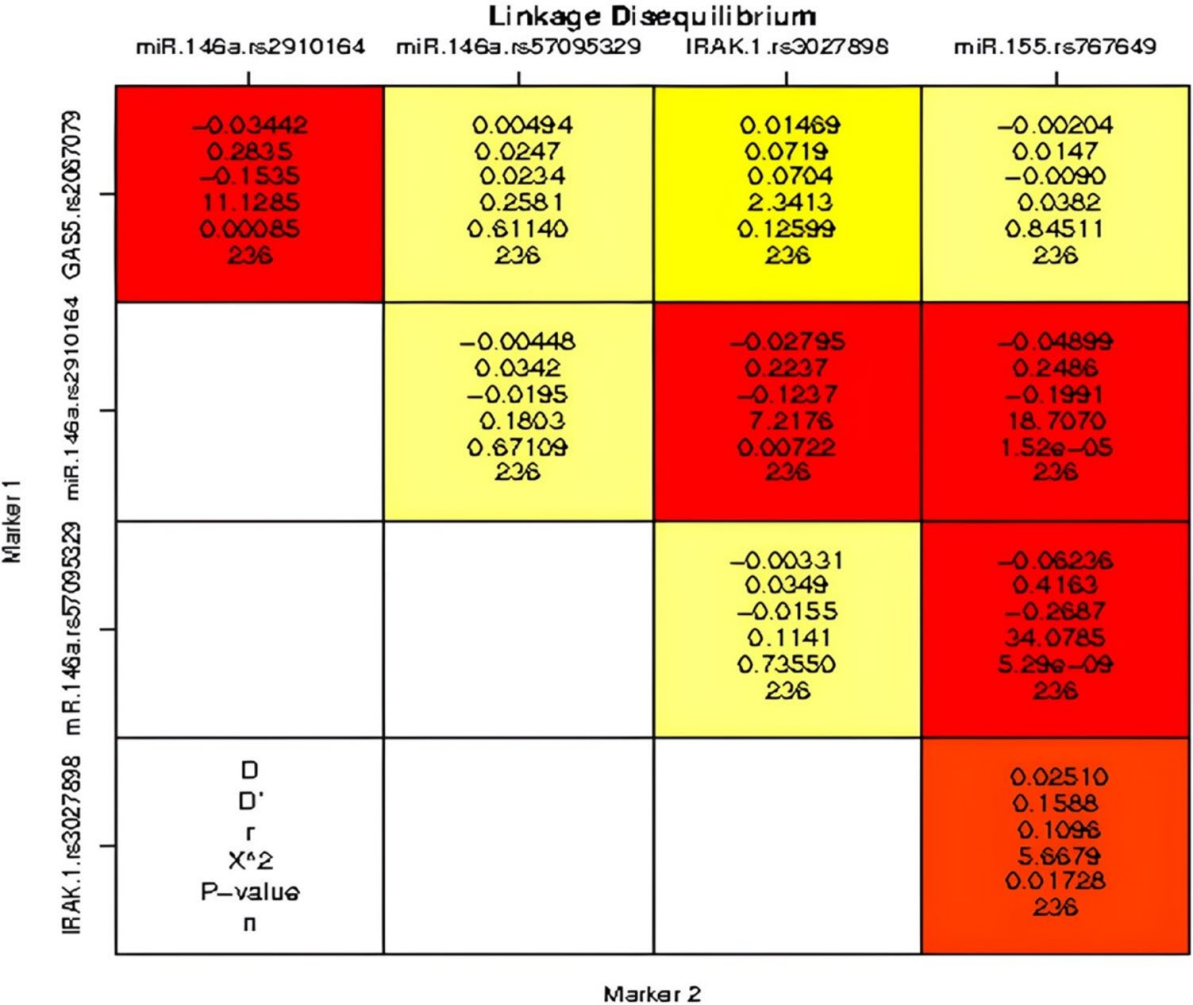
Linkage disequilibrium between the studied SNPs. Linkage disequilibrium was performed in all participants (*n* = 236). The analysis was conducted using SNPStats online software. Red-to-blue scale is presented

We performed a pairwise LD analysis for the studied SNPs (Fig. 1). LD refers to the nonrandom association of alleles at linked loci, typically at the same chromosome, and denotes the probability that two or more loci are co-inherited. However, although rare, LD between different chromosomes has been reported, particularly in cases involving structural variants, chromosomal translocations, or specific genetic interactions [60]. LD was categorized as strong (*D*′ ≥ 0.8), moderate (*D*′ ∼ 0.5), and weak (*D*′ ∼ 0). No strong LD (*D*′ ≥ 0.8) was detected among the investigated SNPs. However, a moderate LD was observed between *miR*-*146a* rs57095329 and *miR-155* rs767649 (*D*′ = 0.416, *P* < 0.0001). On the other hand, a weak LD was found between *GAS5* rs2067079 and *miR*-*146a* rs2910164 (*D*′ = 0.28, *P* = 0.00085) and between *miR*-*146a* rs2910164 and both *IRAK-1* rs3027898 and *miR-155* rs767649 (*D*′ = 0.22, *P* = 0.007 and *D*′ = 0.24, *P* < 0.0001, respectively).

### Association of Epistatic Effects with the Risk of MS and Its Subtypes

This study analyzed the epistatic effects (gene–gene interactions) among five SNPs in association with MS risk (Table 4). To achieve this, we conducted a sub-analysis to examine their joint effects at the genotype level in the overall MS patient and control groups under the codominant model. ORs were adjusted for age and sex as confounding factors.

**Table 4:** Association of epistatic effects (gene–gene interactions) with the risk of MS

	Control	MS	Adjusted OR (95% CI)	Control	MS	Adjusted OR (95% CI)	Control	MS	Adjusted OR (95% CI)
*GAS5* rs2067079 and *miR-146a* rs2910164 cross-classification interaction									
	GG			CG			CC		
CC	13	20	1.00	31	34	0.67 (0.28–1.60)	16	4	**0.10 (0.02–0.43)**
CT	16	13	0.72 (0.25–2.05)	36	22	**0.38 (0.15–0.94)**	4	7	1.42 (0.33–6.07)
TT	3	8	1.96 (0.43–9.00)	1	6	3.23 (0.34–31.18)	0	2	–-
Interaction *P*^*a,#*^-value: 0.0062									
*GAS5* rs2067079 and *miR-146a* rs57095329 cross-classification interaction									
	AA			AG			GG		
CC	22	40	1.00	28	16	**0.32 (0.14–0.73)**	10	2	**0.06 (0.01–0.50)**
CT	7	29	2.48 (0.91–6.74)	39	12	**0.18 (0.08–0.41)**	10	1	**0.07 (0.01–0.61)**
TT	1	12	6.71 (0.81–55.33)	3	3	0.61 (0.11–3.43)	0	1	–-
Interaction *P*^*a*^-value: 0.12									
*GAS5* rs2067079 and *IRAK-1* rs3027898 cross-classification interaction									
	AA			AC			CC		
CC	44	21	1.00	12	27	**4.19 (1.74–10.11)**	4	10	**4.11 (1.11–15.21)**
CT	36	18	1.11 (0.51–2.42)	16	14	1.68 (0.68–4.15)	4	10	**6.41 (1.69–24.32)**
TT	0	6	–-	4	7	3.65 (0.95–14.08)	0	3	–-
Interaction *P*^*a*^-value: 0.027									
*GAS5* rs2067079 and *miR-155* rs767649 cross-classification interaction table									
	AA			AT			TT		
CC	16	9	1.00	37	28	1.51 (0.56–4.04)	7	21	**5.80 (1.72–19.47)**
CT	31	5	0.34 (0.10–1.23)	23	25	2.52 (0.88–7.24)	2	12	**12.06 (2.14–68.02)**
TT	0	0	–-	3	10	**7.28 (1.52–34.93)**	1	6	**11.91 (1.20–118.0)**
Interaction *P*^*a*^-value: 0.15									
*miR-146a* rs2910164 and *miR-146a* rs57095329 cross-classification interaction									
	AA			AG			GG		
GG	2	34	1.00	23	5	**0.01 (0.00–0.07)**	7	2	**0.02 (0.00–0.17)**
CG	20	39	**0.10 (0.02–0.48)**	35	23	**0.04 (0.01–0.17)**	13	0	–-
CC	8	8	**0.05 (0.01–0.29)**	12	3	**0.01 (0.00–0.09)**	0	2	–-
Interaction *P*^*a,#*^-value: < 0.0001									
*miR-146a* rs2910164 and *IRAK-1* rs3027898 cross-classification interaction									
	AA			AC			CC		
GG	19	14	1.00	9	18	2.16 (0.72–6.43)	4	9	2.87 (0.70–11.84)
CG	45	24	0.62 (0.25–1.51)	19	30	1.38 (0.50–3.78)	4	8	1.77 (0.41–7.53)
CC	16	7	0.42 (0.13–1.37)	4	0	0.00	0	6	–-
Interaction *P*^*a*^-value: 0.015									
*miR-146a* rs2910164 and *miR-155* rs767649 cross-classification interaction									
	AA			AT			TT		
GG	8	1	1.00	20	29	**10.92 (1.19–100.6)**	4	11	**20.36 (1.82–227.3)**
CG	27	9	2.35 (0.24–23.35)	35	27	5.55 (0.60–51.40)	6	26	**28.40 (2.70–298.8)**
CC	12	4	1.77 (0.15–20.91)	8	7	5.87 (0.54–63.24)	0	2	–-
Interaction *P*^*a*^-value: 0.45									
*miR-146a* rs57095329 and *IRAK-1* rs3027898 cross-classification interaction									
	AA			AC			CC		
AA	20	36	1.00	6	30	2.77 (0.98–7.84)	4	15	2.03 (0.58–7.03)
AG	48	9	**0.10 (0.04–0.26)**	22	18	0.45 (0.19–1.06)	0	4	–-
GG	12	0	0.00	4	0	0.00	4	4	0.44 (0.08–2.30)
Interaction *P*^*a*^-value: 0.029									
*miR-146a* rs57095329 and *miR-155* rs767649 cross-classification interaction									
	AA			AT			TT		
AA	0	2	1.00	24	54	–-	6	25	–-
AG	40	11	–-	26	6	–-	4	14	–-
GG	7	1	–-	13	3	–-	0	0	–-
Interaction *P*^*a*^-value: 0.015									
*IRAK-1* rs3027898 and *miR-155* rs767649 cross-classification interaction									
	AA			AT			TT		
AA	33	0	1.00	40	35	–-	7	10	–-
AC	14	8	–-	15	21	–-	3	19	–-
CC	0	6	–-	8	7	–-	0	10	–-
Interaction *P*^*a,#*^-value: < 0.0001									

The association analysis was performed using SNPStats online software. All interactions were conducted in the codominant model (control, *n* = 120 vs. MS, *n* = 116). OR and *P*-values in bold are statistically significant, *P* < 0.05. ^a^Adjusted for age and sex in a logistic regression model. ^#^Significant after adjustment for multiple comparisons (Bonferroni *P* < 0.01 threshold)

The results showed significant interactions between *GAS5* rs2067079 and both *miR*-*146a* rs2910164 and *IRAK-1* rs3027898 (*P* < 0.05, before Bonferroni correction). Specifically, the presence of *GAS5* rs2067079-*miR*-*146a* rs2910164 CC + CC and CT + CG genotype combinations conferred protective effects against MS risk, reducing it by approximately 10- and 2.6-fold, respectively (*P* = 0.0062). However, the combination of *GAS5* rs2067079 CC with *IRAK-1* rs3027898 CC or AC conferred at least a fourfold increased risk of MS, while the CT + CC genotype combination of both SNPs conferred a 6.4-fold increased risk.

Beyond *GAS5* rs2067079, *miR*-*146a* rs2910164 exhibited significant interactions with *miR*-*146a* rs57095329 and *IRAK-1* rs3027898 (*P* < 0.05, before Bonferroni correction). Notably, the interaction between *miR*-*146a* rs2910164 and rs57095329 was highly significant (*P* < 0.0001) across several genotype combination levels, conferring a 10- to 100-fold reduced risk of MS.

*miR*-*146a* rs57095329 showed significant interactions with *IRAK-1* rs3027898 and miR-155 rs767649 (*P* < 0.05, before Bonferroni correction). Specifically, the combination of *miR*-*146a* rs57095329 heterozygous AG with *IRAK-1* rs3027898 major homozygous AA conferred a tenfold reduced risk of MS. Additionally, a highly significant interaction was observed between *IRAK-1* rs3027898 and miR-155 rs767649, which was associated with an elevated MS risk (*P* < 0.0001).

Applying a strict Bonferroni correction (*P* < 0.01 threshold) for multiple testing, only the *GAS5* rs2067079-*miR*-*146a* rs2910164, *miR*-*146a* rs2910164-rs57095329, and *IRAK-1* rs3027898-miR-155 rs767649 genetic interactions remained significant. Intriguingly, epistatic interactions exerted stronger effects on MS susceptibility than single-locus effects (Table 4).

Among these interactions, *miR*-*146a* rs2910164-rs57095329 and *IRAK-1* rs3027898-miR-155 rs767649 were the only ones significantly associated with MS risk in RRMS, even after Bonferroni correction (*P* = 0.0081 and < 0.0001, respectively). Specifically, the combination of *miR*-*146a* rs2910164 GG with rs57095329 AG or rs2910164 GG or CC with rs57095329 AA or AG reduced MS risk by several folds (Table 5).

**Table 5:** Association of epistatic effects with the risk of RRMS

	Control	RRMS	Adjusted OR (95% CI)	Control	RRMS	Adjusted OR (95% CI)	Control	RRMS	Adjusted OR (95% CI)
*GAS5* rs2067079 and *miR-146a* rs2910164 cross-classification interaction									
	GG			CG			CC		
CC	13	17	1.00	31	20	0.46 (0.18–1.18)	16	3	**0.11 (0.03–0.48)**
CT	16	9	0.55 (0.18–1.69)	36	18	**0.36 (0.14–0.94)**	4	3	0.51 (0.09–2.76)
TT	3	7	2.12 (0.44–10.1)	1	5	2.62 (0.26–26.40)	0	2	–-
Interaction *P*^*a*^-value: 0.19									
*GAS5* rs2067079 and *miR-146a* rs57095329 cross-classification interaction									
	AA			AG			GG		
CC	22	31	1.00	28	9	**0.24 (0.09–0.61)**	10	0	0.00
CT	7	21	2.32 (0.82–6.62)	39	9	**0.17 (0.07–0.43)**	10	0	0.00
TT	1	11	7.85 (0.94–65.8)	3	3	0.82 (0.14–4.71)	0	0	–-
Interaction *P*^*a*^-value: 0.47									
*GAS5* rs2067079 and *IRAK-1* rs3027898 cross-classification interaction									
	AA			AC			CC		
CC	44	18	1.00	12	17	**3.05 (1.18–7.84)**	4	5	2.57 (0.60–11.00)
CT	36	15	1.09 (0.48–2.48)	16	10	1.34 (0.49–3.65)	4	5	3.57 (0.82–15.55)
TT	0	5	–-	4	7	**4.26 (1.09–16.64)**	0	2	–-
Interaction *P*^*a*^-value: 0.099									
*GAS5* rs2067079 and *miR-155* rs767649 cross-classification interaction table									
	AA			AT			TT		
CC	16	4	1.00	37	21	2.34 (0.69–8.00)	7	15	**8.26 (1.99–34.33)**
CT	31	2	0.27 (0.04–1.63)	23	17	3.61 (0.98–13.32)	2	11	**22.58 (3.44–147.9)**
TT	0	0	–-	3	8	**12.28 (2.12–71.05)**	1	6	**24.71 (2.23–273.67)**
Interaction *P*^*a*^-value: 0.23									
*miR-146a* rs2910164 and *miR-146a* rs57095329 cross-classification interaction									
	AA			AG			GG		
GG	2	28	1.00	23	5	**0.02 (0.00–0.09)**	7	0	0.00
CG	20	28	**0.09 (0.02–0.44)**	35	15	**0.03 (0.01–0.14)**	13	0	0.00
CC	8	7	**0.05 (0.01–0.32)**	12	1	**0.01 (0.00–0.06)**	0	0	–-
Interaction *P*^*a,#*^-value: 0.0081									
*miR-146a* rs2910164 and *IRAK-1* rs3027898 cross-classification interaction									
	AA			AC			CC		
GG	19	13	1.00	9	15	1.94 (0.63–6.00)	4	5	1.98 (0.42–9.28)
CG	45	18	0.51 (0.20–1.31)	19	19	0.95 (0.32–2.81)	4	6	1.40 (0.30–6.51)
CC	16	7	0.47 (0.14–1.55)	4	0	0.00	0	1	–-
Interaction *P*^*a*^-value: 0.2									
*miR-146a* rs2910164 and *miR-155* rs767649 cross-classification interaction									
	AA			AT			TT		
GG	8	1	1.00	20	21	7.92 (0.84–74.38)	4	11	**19.95 (1.77–224.91)**
CG	27	5	1.24 (0.11–13.61)	35	19	3.85 (0.40–36.87)	6	19	**18.96 (1.74–207.17)**
CC	12	0	0.00	8	6	4.48 (0.40–50.60)	0	2	–-
Interaction *P*^*a*^-value: 0.27									
*miR-146a* rs57095329 and *IRAK-1* rs3027898 cross-classification interaction									
	AA			AC			CC		
AA	20	29	1.00	6	23	2.79 (0.95–8.18)	4	11	2.08 (0.56–7.71)
AG	48	9	**0.12 (0.05–0.31)**	22	11	**0.38 (0.15–0.99)**	0	1	–-
GG	12	0	0.00	4	0	0.00	4	0	0.00
Interaction *P*^*a*^-value: 0.62									
*miR-146a* rs57095329 and *miR-155* rs767649 cross-classification interaction									
	AA			AT			TT		
AA	0	1	1.00	24	41	0.00	6	21	0.00
AG	40	5	0.00	26	5	0.00	4	11	0.00
GG	7	0	0.00	13	0	0.00	0	0	–-
Interaction *P*^*a*^-value: 0.089									
*IRAK-1* rs3027898 and *miR-155* rs767649 cross-classification interaction									
	AA			AT			TT		
AA	33	0	1.00	40	28	–-	7	10	–-
AC	14	5	–-	15	16	–-	3	13	–-
CC	0	1	–-	8	2	–-	0	9	–-
Interaction *P*^*a,#*^-value: 4e-04									

The association analysis was performed using SNPStats online software. All interactions were conducted in the codominant model (control, *n* = 120 vs. RRMS, *n* = 84). OR and *P*-values in bold are statistically significant, *P* < 0.05. ^a^Adjusted for age and sex in a logistic regression model. ^#^Significant after adjustment for multiple comparisons (Bonferroni *P* < 0.01 threshold)

Table 6 presents the associations of SNP-SNP interactions with SPMS risk. Interestingly, SPMS patients exhibited the same pattern of SNP-SNP interactions observed in the overall MS group compared to healthy controls. Additional interactions were identified between *GAS5* rs2067079 and *miR*-*146a* rs57095329, as well as between *miR*-*146a* rs2910164 and miR-155 rs767649, in association with SPMS risk (*P* = 0.047 and 0.022, respectively); however, these associations were non-significant after Bonferroni correction.

**Table 6:** Association of epistatic effects with the risk of SPMS

	Control	SPMS	Adjusted OR (95% CI)	Control	SPMS	Adjusted OR (95% CI)	Control	SPMS	Adjusted OR (95% CI)
*GAS5* rs2067079 and *miR-146a* rs2910164 cross-classification interaction									
	GG			CG			CC		
CC	13	3	1.00	31	14	1.94 (0.45–8.28)	16	1	0.23 (0.02–2.50)
CT	16	4	1.95 (0.32–11.73)	36	4	0.42 (0.08–2.29)	4	4	7.42 (1.00–54.97)
TT	3	1	1.20 (0.08–17.89)	1	1	3.99 (0.15–102.77)	0	0	–-
Interaction *P*^*a,#*^-value: 0.0012									
*GAS5* rs2067079 and *miR-146a* rs57095329 cross-classification interaction									
	AA			AG			GG		
CC	22	9	1.00	28	7	0.64 (0.20–2.03)	10	2	0.54 (0.10–3.03)
CT	7	8	3.01 (0.77–11.72)	39	3	**0.20 (0.05–0.84)**	10	1	0.30 (0.03–3.11)
TT	1	1	2.45 (0.13–45.09)	3	0	0.00	0	1	–-
Interaction *P*^*a*^-value: 0.047									
*GAS5* rs2067079 and *IRAK-1* rs3027898 cross-classification interaction									
	AA			AC			CC		
CC	44	3	1.00	12	10	**9.18 (2.04–41.41)**	4	5	**15.28 (2.57–90.7)**
CT	36	3	1.39 (0.26–7.48)	16	4	3.02 (0.58–15.75)	4	5	**40.69 (4.21–393.6)**
TT	0	1	–-	4	0	0.00	0	1	–-
Interaction *P*^*a*^-value: 0.015									
*GAS5* rs2067079 and *miR-155* rs767649 cross-classification interaction table									
	AA			AT			TT		
CC	16	5	1.00	37	7	0.57 (0.15–2.08)	7	6	2.56 (0.58–11.38)
CT	31	3	0.33 (0.07–1.60)	23	8	1.26 (0.32–4.90)	2	1	1.38 (0.10–19.12)
TT	0	0	–-	3	2	2.32 (0.27–20.10)	1	0	0.00
Interaction *P*^*a*^-value: 0.11									
*miR-146a* rs2910164 and *miR-146a* rs57095329 cross-classification interaction									
	AA			AG			GG		
GG	2	6	1.00	23	0	0.00	7	2	0.11 (0.01–1.12)
CG	20	11	**0.15 (0.02–0.91)**	35	8	**0.07 (0.01–0.40)**	13	0	0.00
CC	8	1	**0.03 (0.00–0.42)**	12	2	**0.05 (0.00–0.44)**	0	2	–-
Interaction *P*^*a,#*^-value: < 0.0001									
*miR-146a* rs2910164 and *IRAK-1* rs3027898 cross-classification interaction									
	AA			AC			CC		
GG	19	1	1.00	9	3	5.41 (0.43–68.01)	4	4	**27.04 (1.97–371.88)**
CG	45	6	2.58 (0.26–25.39)	19	11	7.46 (0.72–77.00)	4	2	7.57 (0.52–111.22)
CC	16	0	0.00	4	0	0.00	0	5	–-
Interaction *P*^*a,#*^-value: 0.0023									
*miR-146a* rs2910164 and *miR-155* rs767649 cross-classification interaction									
	AA			AT			TT		
GG	8	0	1.00	20	8	–-	4	0	0.89
CG	27	4	–-	35	8	–-	6	7	–-
CC	12	4	–-	8	1	–-	0	0	–-
Interaction *P*^*a*^-value: 0.022									
*miR-146a* rs57095329 and *IRAK-1* rs3027898 cross-classification interaction									
	AA			AC			CC		
AA	20	7	1.00	6	7	3.69 (0.85–16.06)	4	4	2.66 (0.51–13.87)
AG	48	0	0.00	22	7	0.90 (0.26–3.16)	0	3	–-
GG	12	0	0.00	4	0	0.00	4	4	3.94 (0.66–23.40)
Interaction *P*^a,#^-value: 0.0012									
*miR-146a* rs57095329 and *miR-155* rs767649 cross-classification interaction									
	AA			AT			TT		
AA	0	1	1.00	24	13	0.00	6	4	0.00
AG	40	6	0.00	26	1	0.00	4	3	0.00
GG	7	1	0.00	13	3	0.00	0	0	–-
Interaction *P*^*a*^-value: 0.041									
*IRAK-1* rs3027898 and *miR-155* rs767649 cross-classification interaction									
	AA			AT			TT		
AA	33	0	1.00	40	7	–-	7	0	0.81
AC	14	3	–-	15	5	–-	3	6	–-
CC	0	5	–-	8	5	–-	0	1	–-
Interaction *P*^*a,#*^-value: 0.00044									

The association analysis was performed using SNPStats online software. All interactions were carried out in the codominant model (control, *n* = 120 vs. SPMS, *n* = 32). OR and *P*-values in bold are statistically significant, *P* < 0.05. ^a^Adjusted for age and sex in a logistic regression model. ^#^Significant after adjustment for multiple comparisons (Bonferroni *P* < 0.01 threshold)

After applying a strict Bonferroni correction (*P* < 0.01), only five genetic interactions remained significantly associated with SPMS risk: *GAS5* rs2067079-*miR*-*146a* rs2910164, *miR*-*146a* rs2910164-rs57095329, *miR*-*146a* rs2910164-*IRAK-1* rs3027898, *miR*-*146a* rs57095329-*IRAK-1* rs3027898, and *IRAK-1* rs3027898-*miR-155* rs767649.

### Risk Stratification Analysis of Gene–Gene Interactions by Gender

We first assessed the association of single-locus effects with MS risk using the codominant model in risk-stratified groups by sex, given that female sex is a major MS risk factor (Supplementary Table S7). Only *miR*-*146a* rs2910164 and *miR-155* rs767649 exhibited significant gender-specific associations (*P* < 0.0001 and *P* = 0.027, respectively). The minor CC and heterozygous CG genotypes of *miR*-*146a* rs2910164 were protective against MS risk in females (CC vs. GG, adjusted OR (95% CI) = 0.23 (0.07–0.73)) and males (CG vs. GG, adjusted OR (95% CI) = 0.17 (0.06–0.50)), respectively, compared to their corresponding controls. Conversely, the AT or TT genotypes of *miR-155* rs767649 were associated with an increased MS risk in females compared to controls (AT vs. AA and TT vs. AA, adjusted OR (95% CI) = 4.88 (1.27–18.73) and 10.98 (2.55–47.32), respectively).

We then tested the association of SNP-SNP interactions within each gender separately in cases and corresponding controls with MS risk. After multiple comparison adjustments (Bonferroni *P* < 0.01), five significant SNP-SNP interactions were associated with MS risk in females (Supplementary Table S8). Notably, four interactions followed the same pattern as those associated with overall MS risk. The *miR*-*146a* rs2910164-rs57095329 and *miR*-*146a* rs57095329-*IRAK-1* rs3027898 interactions were protective, whereas *miR*-*146a* rs2910164-*IRAK-1* rs3027898 and *IRAK-1* rs3027898-*miR-155* rs767649 were significantly associated with an increased MS risk in females. Additionally, *GAS5* rs2067079 significantly interacted with *miR-155* rs767649, increasing MS risk.

In males, only *miR*-*146a* rs2910164-*IRAK-1* rs3027898 and *IRAK-1* rs3027898-*miR-155* rs767649 SNP-SNP interactions contributed to an increased MS risk after multiple comparison adjustments (Bonferroni *P* < 0.01) (Supplementary Table S9).

### Association of Allelic Combinations with the Susceptibility to MS Through a Haplotype Analysis

A haplotype analysis of the five studied SNPs identified seven different allelic combinations with total pooled frequencies of MS patients and healthy controls ≥ 0.05 (Table 7). Testing haplotype associations with MS risk revealed that the CCAAA haplotype (alleles in the order of *GAS5* rs2067079, *miR*-*146a* rs2910164, rs57095329, *IRAK-1* rs3027898, and *miR-155* rs767649) conferred protection against MS risk compared to controls (CCAAA vs. CGAAT, adjusted OR = 0.14, 95% CI = 0.03–0.69, *P* = 0.009) after adjustment for demographic variables. The significance persisted after setting a strict Bonferroni correction (*P* < 0.01).

**Table 7:** Association of haplotypes with the risk of MS

	*GAS5* rs2067079	*miR-146a* rs2910164	*miR-146a* rs57095329	*IRAK-1* rs3027898	*miR-155* rs767649	Total frequency	Adjusted OR (95% CI)	*P*^a^-value
1	C	G^*^	A^*^	A	T^*^	0.1079	1.00	–-
2	C	C	A^*^	A	T^*^	0.0862	0.49 (0.05–4.40)	0.52
3	T^*^	G^*^	A^*^	A	A	0.0852	0.65 (0.13–3.39)	0.61
4	C	C	A^*^	A	A	0.0849	**0.14 (0.03–0.69)**	**0.009**
5	C	G^*^	G	A	A	0.0833	0.00	1
6	C	C	G	A	A	0.0573	0.00	1
7	T^*^	G^*^	A^*^	C^*^	T^*^	0.0549	2.44 (0.23–26.31)	0.46
8	C	G^*^	A^*^	C^*^	A	0.0389	0.47 (0.10–2.30)	0.35
Global haplotype association *P*^*a,#*^-value: < 0.0001								

The haplotype analysis was performed using SNPStats online software. ^*^Indicates the risk allele. Total frequency threshold for rare haplotypes was adjusted at 0.05. The haplotype with the highest total (pooled) frequency from controls (*n* = 120) and cases (*n* = 116) was set as a reference. Bold indicates statistical significance, *P* < 0.05. ^a^Adjusted for age and sex in a logistic regression model. ^#^Significant after adjustment for multiple comparisons (Bonferroni *P* < 0.01 threshold)

### Association of Distinct SNPs with MS Severity Among MS Patients

Using the best-fit model approach, we examined the association of each SNP with MS severity, represented by the EDSS, where MS patients were stratified into less severe (EDSS < 6) and most severe (EDSS ≥ 6) groups (Table 8). Three of the five SNPs (*miR*-*146a* rs57095329, *IRAK-1* rs3027898, and *miR-155* rs767649) showed a significant positive association with severe MS (EDSS ≥ 6). *miR*-*146a* rs57095329 was best associated with EDSS ≥ 6 in the log-additive model (adjusted OR (95% CI) = 2.82 (1.32–6.03), *P* = 0.0071). Notably, MS patients with EDSS ≥ 6 had a higher frequency of the minor homozygous CC genotype of *IRAK-1* rs3027898 (CC vs. AA + AC, adjusted OR (95% CI) = 4.53 (1.68–12.22), *P* = 0.0031) and the major homozygous AA genotype of *miR-155* rs767649 (AA vs. TT + AT, adjusted OR = 4.35 (1.35–14.06), *P* = 0.016).

**Table 8:** Association of distinct SNPs with MS severity among MS patients

SNP	Model	Genotype	EDSS < 6 (*n* = 89)	EDSS ≥ 6 (*n* = 27)	Adjusted OR (95% CI)	*P*^a^-value
*GAS5* rs2067079	Recessive	CC + CT	75 (84.3%)	25 (92.6%)	1.00	0.23
		TT	14 (15.7%)	2 (7.4%)	0.41 (0.09–1.97)	
*miR-146a* rs2910164	Dominant	G/G	34 (38.2%)	7 (25.9%)	1.00	0.18
		CG + CC	55 (61.8%)	20 (74.1%)	1.94 (0.72–5.24)	
*miR-146a* rs57095329	Log-additive	–-	–-	–-	**2.82 (1.32–6.03)**	**0.0071**
*IRAK-1* rs3027898	Recessive	AA + AC	77 (86.5%)	16 (59.3%)	1.00	**0.0031**
		CC	12 (13.5%)	11 (40.7%)	**4.53 (1.68–12.22)**	
*miR-155* rs767649	Recessive	TT + AT	82 (92.1%)	20 (74.1%)	1.00	**0.016**
		AA	7 (7.9%)	7 (25.9%)	**4.35 (1.35–14.06)**	
			**RRMS (*n* = 84)**	**SPMS (*n* = 32)**		
*GAS5* rs2067079	Recessive	CC + CT	70 (83.3%)	30 (93.8%)	1.00	0.11
		TT	14 (16.7%)	2 (6.2%)	0.32 (0.07–1.53)	
*miR-146a* rs2910164	Dominant	GG	33 (39.3%)	8 (25%)	1.00	0.11
		CG + CC	51 (60.7%)	24 (75%)	2.10 (0.82–5.36)	
*miR-146a* rs57095329	Log-additive	–-	–-	–-	**2.71 (1.30–5.67)**	**0.0071**
*IRAK-1* rs3027898	Dominant	AA	38 (45.2%)	7 (21.9%)	1.00	**0.012**
		AC + CC	46 (54.8%)	25 (78.1%)	**3.31 (1.24–8.84)**	
*miR-155* rs767649	Recessive	TT + AT	78 (92.9%)	24 (75%)	1.00	**0.01**
		AA	6 (7.1%)	8 (25%)	**4.53 (1.41–14.51)**	

The best fit model is shown. The association analysis was performed using SNPStats online software. The genotype(s) with the highest frequency was set as a reference. ^a^Adjusted for age and sex in a logistic regression model. *P*-values in bold are statistically significant, *P* < 0.05

Similarly, these SNPs were associated with the presence of SPMS among MS patients (Table 8). Specifically, *miR*-*146a* rs57095329 was best associated with the presence of SPMS in the log-additive model (adjusted OR (95% CI) = 2.71 (1.30–5.67), *P* = 0.0071). Interestingly, SPMS was more prevalent than RRMS in MS patients carrying the AC + CC genotypes of *IRAK-1* rs3027898 (AC + CC vs. AA, adjusted OR (95% CI) = 3.31 (1.24–8.84), *P* = 0.012) or the AA genotype of *miR-155* rs767649 (AA vs. TT + AT, adjusted OR (95% CI) = 4.53 (1.41–14.51), *P* = 0.01).

### Association of Haplotypes with the Demographic and Clinical Data Among MS Patients

We tested the association of haplotypes with sex, age, EDSS, and MS type among MS patients (Table 9). The results revealed a significant global interaction of haplotypes with female gender, EDSS, and MS type (*P* = 0.006, 0.0033, and 0.0048, respectively) (Bonferroni *P* < 0.01). Specifically, female MS patients exhibited a significantly higher frequency of the CGACT haplotype (referring to alleles of rs2067079, rs2910164, rs57095329, rs3027898, and rs767649) than males (CGACT vs. CGAAA, adjusted OR (95%CI) = 27.63 (1.51–507.12), *P* = 0.028). In contrast, the CGAAT haplotype, which contains the protective A allele of *IRAK-1* rs3027898, was 25 times more prevalent in male MS patients than females (CGAAT vs. CGAAA, adjusted OR (95% CI) = 0.04 (0.00–0.73), *P* = 0.032).

**Table 9:** Association of haplotypes with the demographic and clinical data among MS patients

*GAS5* rs2067079	*miR-146a* rs2910164	*miR-146a* rs57095329	*IRAK-1* rs3027898	*miR-155* rs767649	Total frequency	Frequency in males (*n* = 32)	Frequency in females (*n* = 84)	Adjusted OR (95% CI)	*P*-value
C	G^*^	A^*^	A	A	0.145	0.157	0.1397	1.00	
C	C	A^*^	A	T^*^	0.1203	0.0977	0.1509	17.96 (0.97–332.57)	0.055
C	G^*^	A^*^	C^*^	T^*^	0.0912	0.0752	0.1117	**27.63 (1.51–507.12)**	**0.028**
C	G^*^	A^*^	A	T^*^	0.0893	0.1323	0.0606	**0.04 (0.00–0.73)**	**0.032**
T^*^	G^*^	A^*^	C^*^	T^*^	0.0734	0	0.0527	0.81 (0.13–5.10)	0.82
T^*^	G^*^	A^*^	A	A	0.0649	NA	0.0652	1.37 (0.14–13.59)	0.79
C	C	G	C*	A	0.064	NA	0.0636	0.95 (0.11–8.31)	0.96
Global haplotype association *P*^*a,#*^-value: 0.006									
						**Age < 30 (*n* = 56)**	**Age ≥ 30 (*n* = 60)**		
C	G*	A*	A	A	0.141	0.1359	0.1282	1.00	
C	G*	A*	C*	T*	0.1172	0.1849	0.0446	**0.20 (0.04–0.92)**	**0.041**
C	C	A*	A	T*	0.1065	0.0938	0.1345	0.93 (0.15–5.56)	0.93
C	G*	A*	A	T*	0.0808	0.0346	0.1462	2.52 (0.30–21.36)	0.4
T*	G*	A*	C*	T*	0.0678	0	0.0747	0.67 (0.14–3.33)	0.63
C	C	G	C*	A	0.0655	0.0654	0.0673	0.61 (0.11–3.49)	0.58
T*	G*	A*	A	A	0.0584	0.0153	0.0676	0.91 (0.13–6.33)	0.92
Global haplotype association *P*^b^-value: 0.19									
GAS5 rs2067079	miR-146a rs2910164	miR-146a rs57095329	IRAK-1 rs3027898	miR-155 rs767649	Total frequency	Frequency in EDSS < 6 (*n* = 89)	Frequency in EDSS ≥ 6 (*n* = 27)	Adjusted OR (95% CI)	***P***^**a**^**-value**
C	G^*^	A^*^	A	A	0.1491	0.1346	0.1862	1.00	–-
C	G^*^	A^*^	C^*^	T^*^	0.1078	0.1289	0.0619	0.99 (0.17–5.64)	0.99
C	C	A^*^	A	T^*^	0.101	0.0889	0.1257	1.85 (0.24–14.24)	0.56
C	G^*^	A^*^	A	T^*^	0.0821	0.1109	NA	0.00	1
T*	G^*^	A^*^	C^*^	T^*^	0.075	0.0755	0.0797	1.57 (0.28–8.71)	0.61
C	C	G	C^*^	A	0.065	0.0326	0.1744	**9.23 (1.05–80.86)**	**0.047**
T*	G^*^	A^*^	A	A	0.0491	0.0665	0.0287	0.00	1
Global haplotype association *P*^*c,#*^-value: 0.0033									
						**Frequency in RRMS (*n* = 84)**	**Frequency in SPMS (*n* = 32)**		
C	G^*^	A^*^	A	A	0.142	0.1347	0.1649	1.00	–-
C	G*	A^*^	C^*^	T^*^	0.1129	0.1138	0.0923	2.27 (0.34–15.15)	0.4
C	C	A^*^	A	T^*^	0.1006	0.0869	0.1697	2.07 (0.19–22.11)	0.55
C	G^*^	A^*^	A	T^*^	0.0838	0.1161	NA	0.00	1
T^*^	G^*^	A^*^	C^*^	T^*^	0.073	0.0849	0.0437	0.62 (0.07–5.27)	0.66
C	C	G	C^*^	A	0.0647	0.0346	0.1454	6.20 (0.75–51.53)	0.094
T^*^	G^*^	A^*^	A	A	0.0597	0.0697	0.0428	0.47 (0.01–20.88)	0.7
Global haplotype association *P*^*c,#*^-value: 0.0048									

The haplotype analysis was performed using SNPStats online software. ^*^Indicates the risk allele. Total frequency threshold for rare haplotypes was adjusted at 0.05. The haplotype with the highest total (pooled) frequency was set as a reference. The results are adjusted with ^a^ age, ^b^ sex, and ^c^ age and sex in a logistic regression model. *NA*, not available. Bold indicates statistical significance, *P* < 0.05. ^#^Significant after adjustment for multiple comparisons (Bonferroni *P* < 0.01 threshold)

Regarding age, younger MS patients (< 30 years) harbored the CGACT haplotype five times more frequently than older patients (≥ 30 years) (CGACT vs. CGAAA, adjusted OR (95% CI) = 0.20 (0.04–0.92), *P* = 0.041); however, the global interaction for age was non-significant (*P* = 0.19).

MS patients with EDSS ≥ 6 had a significantly higher frequency of the CCGCA haplotype than those with EDSS < 6 (CCGCA vs. CGAAA, adjusted OR (95%CI) = 9.23 (1.05–80.86), *P* = 0.047). The same haplotype was more common in SPMS than in RRMS patients. Although the individual association of this haplotype with MS type did not reach statistical significance (*P* = 0.094), the global interaction was significant (*P* = 0.0048).

### Results of eQTL Analysis

**Fig. 2:**
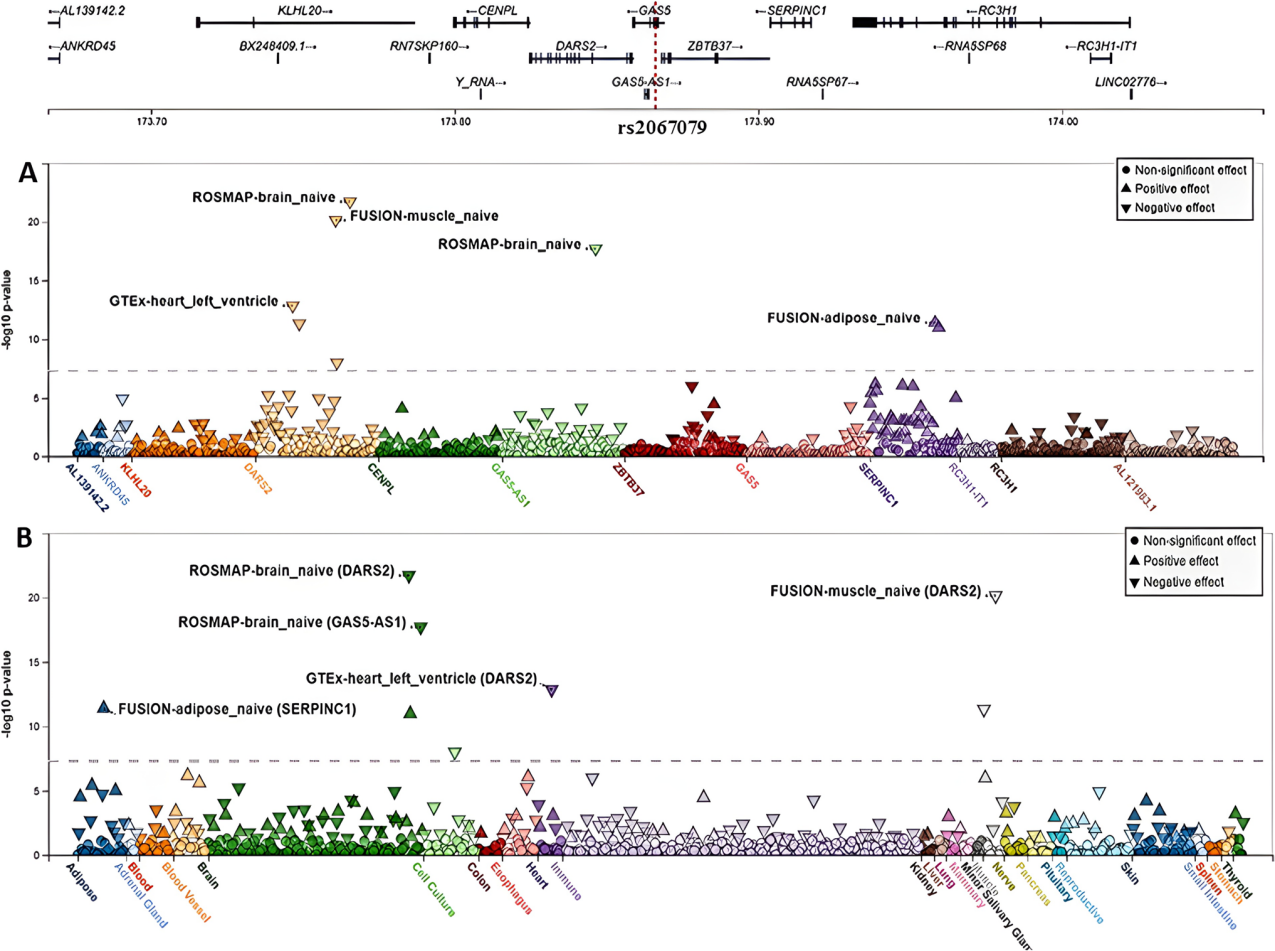
cis-eQTLs associated with variant rs2067079 (1:173,866,073_C/T). **A** A variant-centric view of eQTL *P*-values for rs2067079. This variant is a strong cis-eQTL regulating multiple genes: DARS2 (− log_10_ *P*-value = 21.72, effect size = − 0.15, negative effect) and *GAS5-AS1* in the brain (− log_10_ *P*-value = 17.71, effect size = − 0.26, negative effect). **B** The same view showing the signals in different systems, with a strong signal appearing for *DARS2* and *GAS5-AS1* in the brain. Data were generated from the FIVEx browser (https://fivex.sph.umich.edu/). DARS2, aspartyl-tRNA synthetase 2; GAS5-AS1, growth-arrest specific transcript 5-antisense 1

**Fig. 3:**
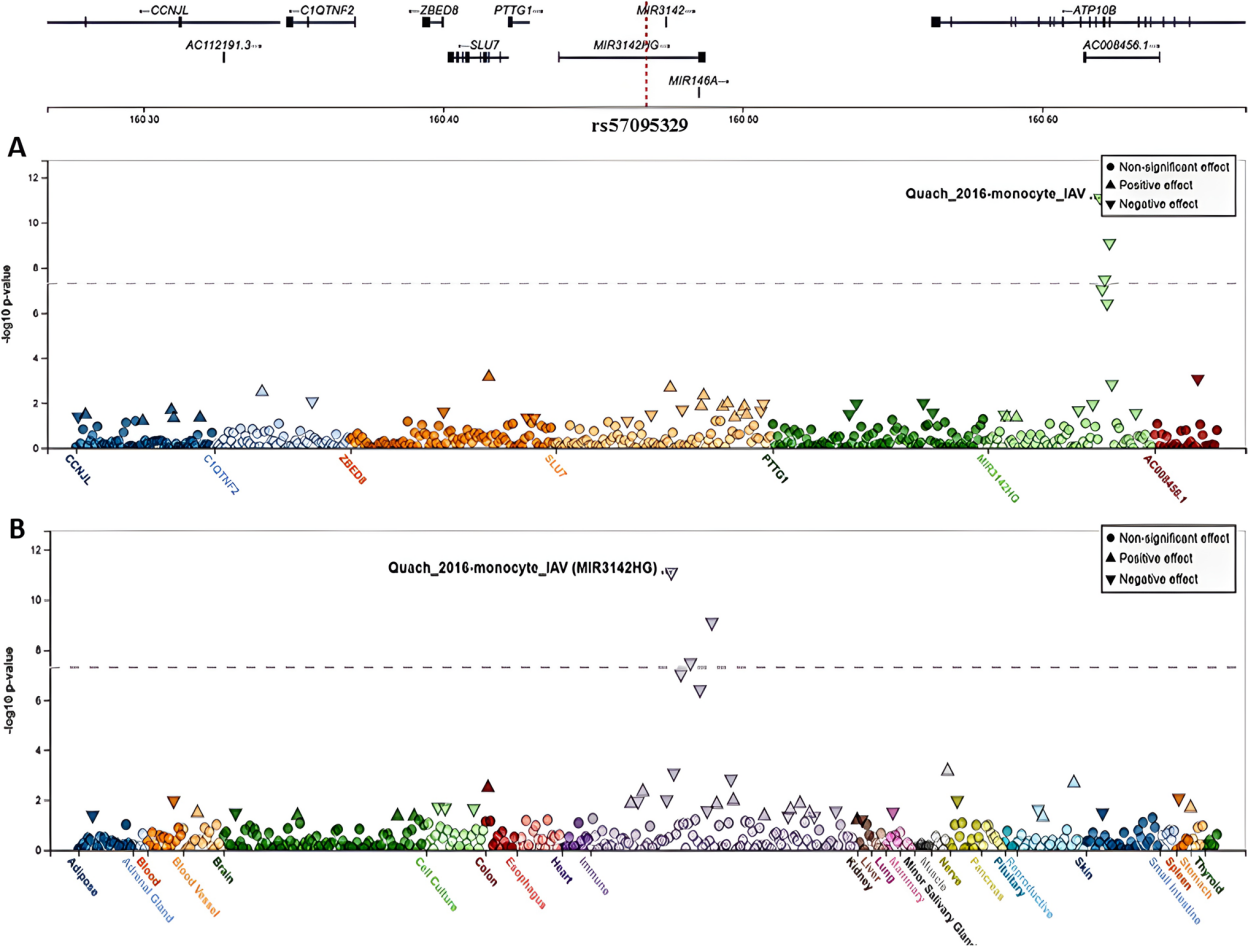
cis-eQTLs associated with variant rs57095329 (5:160,467,840_A/G). **A** A variant-centric view of eQTL *P*-values for rs57095329. This variant is a strong cis-eQTL regulating *MIR3142HG* in monocytes (− log_10_ *P*-value = 11.09, effect size = − 0.53, negative effect). **B** The same view showing the signals in different systems, with a strong signal for *MIR3142HG* appearing in monocytes. Data were generated from the FIVEx browser (https://fivex.sph.umich.edu/)

**Fig. 4:**
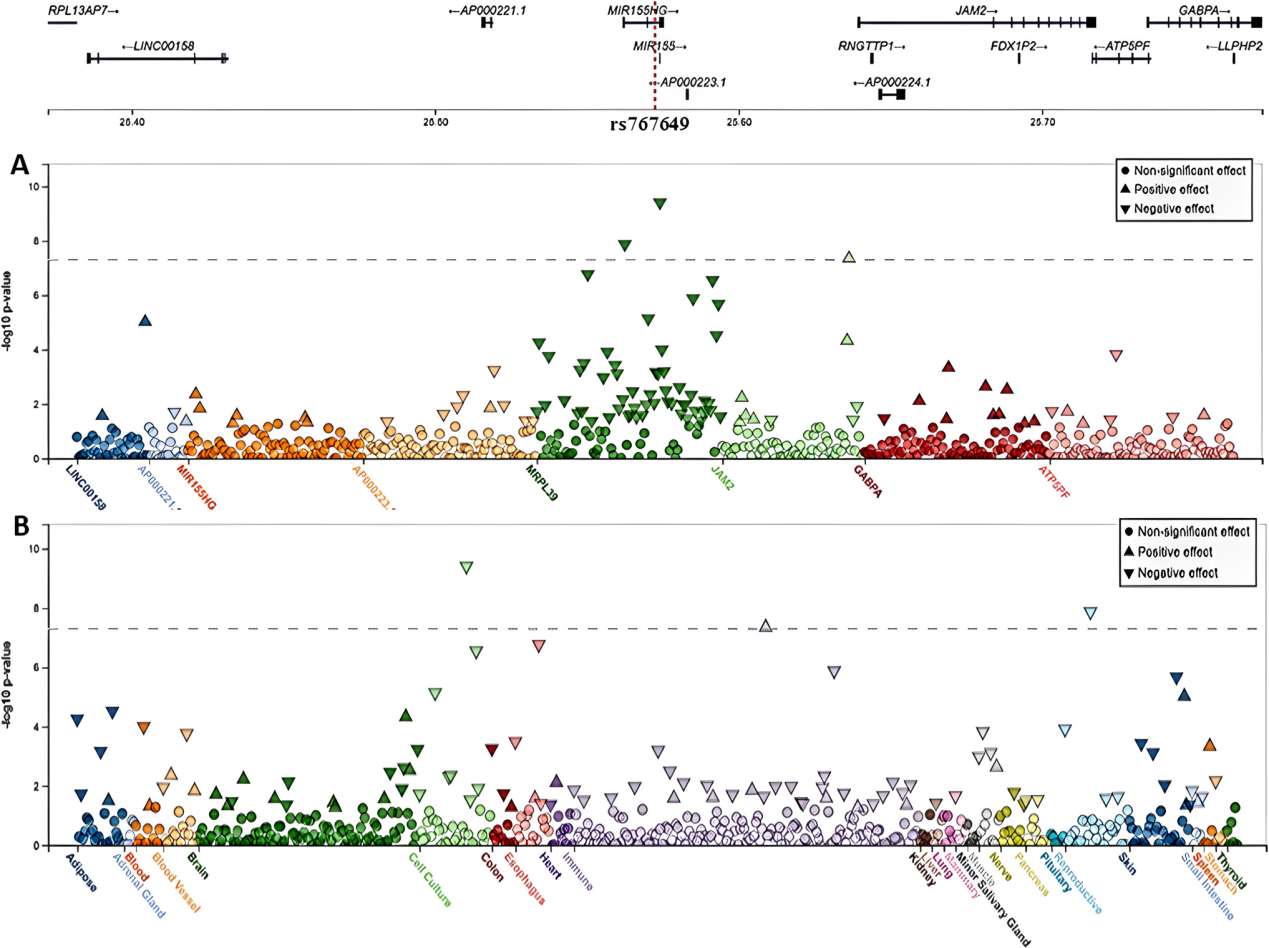
cis-eQTLs associated with variant: rs767649 (21:25,572,410_T/A). **A** A variant-centric view of eQTL *P*-values for rs767649. This variant has a feature of a strong cis-eQTL regulating *JAM2* and *MRPL39*. **B** The same view showing the signals in different systems, with a strong signal for *JAM2* appears in B-cells (− log_10_ *P*-value = 7.36, effect size = 0.74, positive effect) and *MRPL39* in cell culture, testis, and memory T follicular helper (Tfh-memory) CD4 + T cells (− log_10_ *P*-value = 5.88, effect size = − 0.24, negative effect). Data were generated from the FIVEx browser (https://fivex.sph.umich.edu/). JAM2, junctional adhesion molecule 2; MRPL39, mitochondrial ribosomal protein L39

We contemplated the cis-eQTLs associated with the tested genetic variants to elucidate their potential tissue-specific regulatory effects, particularly in the brain, immune system, and whole blood, which are primarily involved in MS. Notably, three of the five variants examined in this study are strong cis-eQTLs (passing the eQTL − log_10_ *P*-value threshold) according to the FIVEx browser (Figs. 2, 3, 4). The rs2067079 variant is a strong cis-eQTL regulating multiple genes, with the top two being *DARS2* (aspartyl-tRNA synthetase 2) and *GAS5*-*AS1* (*GAS5*-antisense 1), showing strong signals in the brain (Fig. 2). Locus-centric views of single-tissue eQTLs near *DARS2* and *GAS5*-*AS1* in the brain are presented in Supplementary Figure S1.

The rs57095329 variant is a strong eQTL regulating *MIR3142HG*, the host gene of *miR*-*146a*, in monocytes (Fig. 3). The FIVEx browser provides the posterior inclusion probability (PIP), a ranking measure indicating how likely a variable is to be included in the true regression model, thus suggesting its potential causality [61]. Notably, the PIP for rs57095329 affecting *MIR3142HG* is 1, indicating that this variant might be causal (Supplementary Figure S2).

**Fig. 5:**
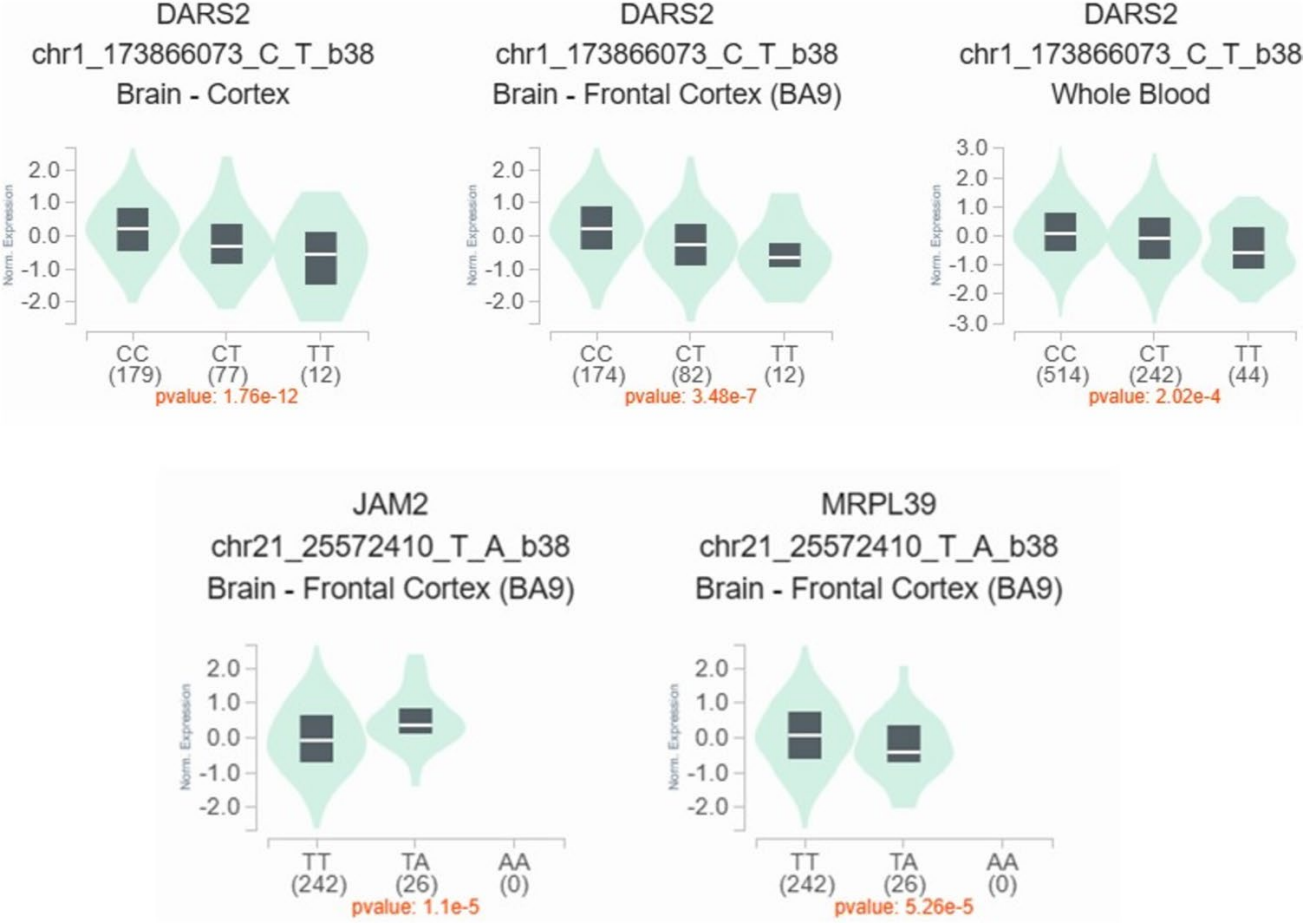
Violin plots of single-tissue cis-eQTLs associated with rs2067079 (1_173866073_C/T) and rs767649 (21_25572410_T/A) in MS-relevant tissues. Data were generated from the GTEx portal browser (GTEx Analysis Release V10) (https://www.gtexportal.org/home/). DARS2, aspartyl-tRNA synthetase 2; JAM2, junctional adhesion molecule 2; MRPL39, mitochondrial ribosomal protein L39

Additionally, the rs767649 variant exhibits strong eQTL features for *JAM2* (junctional adhesion molecule 2) and *MRPL39* (mitochondrial ribosomal protein L39) (Fig. 4). A strong signal of rs767649 for *JAM2* was observed in B cells. Locus-centric views of single-tissue eQTLs near the *MIR3142HG* and *JAM2* regions in monocytes and B cells, respectively, are visualized in Supplementary Figures S2 and S3. The cis-eQTL effects of rs2067079 and rs767649 were further validated using the eQTLGen phase I database. Violin plots of single-tissue cis-eQTLs for rs2067079 on *DARS2* and rs767649 on *JAM2* and *MRPL39* in MS-relevant tissues (brain and whole blood) from the GTEx portal are shown in Fig. 5.

Notably, the rs2910164 variant did not exhibit a strong cis-eQTL effect for nearby genes across multiple systems (Supplementary Figure S4), while rs3027898 was not reported in the eQTL databases searched.

We explored the trans-eQTL effects of the tested SNPs. According to eQTLGen phase I, none of the five investigated SNPs exhibited trans-eQTL effects. However, in the ncRNA-eQTL database, only the rs2067079 variant demonstrated a trans-eQTL effect on *RP11*-*713C19.2* (ENSG00000213331.4, *PRDX6*, peroxiredoxin 6, processed pseudogene) in three cancer types (breast cancer, head and neck carcinoma, and hepatocellular carcinoma) (Supplementary Figure S5).

## Discussion

MS is a multifactorial polygenic disease, yet much of its genetic complexity remains unidentified. To our knowledge, this study provides the first evidence of epistatic, or gene–gene interactions associated with MS susceptibility in the Egyptian population. Although the selected SNPs have not been reported by the International Multiple Sclerosis Genetics Consortium (IMSGC), some have been linked to MS risk and severity in more extensive studies across different populations [32–34]. We recapitulated their single-locus effects and identified several significant genetic interactions concerning the susceptibility to MS and its phenotypes between multiple loci. These interactions contributed to an amplified risk beyond the effects observed at individual loci.

The same pattern of SNP-SNP interactions, as observed in association with MS risk, was specific to females. Likewise, gender-specific associations of miRNA genetic variant combinations were previously reported in a Russian MS ethnic group [34]. Given that the female gender is a well-established risk factor for MS, the presence of these particular gene–gene interactions may help explain the genetic basis of sex differences in MS susceptibility.

Furthermore, haplotype analysis revealed multiple allelic combinations of the studied SNPs that may predispose individuals to MS, associate with female gender, and correlate with disease severity. These findings highlight the crucial role of epistatic interactions and haplotypes in MS development and progression.

More specifically, our findings figured out an interaction between a genetic variant of *GAS5* and its putative target axis, *miR*-*146a/IRAK-1*. These interactions were particularly significant in the SPMS group, suggesting their potential involvement in MS severity. This may partly be attributed to an altered expression of the *GAS5/miR*-*146a/IRAK-1* axis.

The mechanistic relationship between *GAS5* rs2067079 and MS predisposition was elucidated in our previous study, where C allele-associated genotypes correlated with lower serum *GAS5* levels [29]. In contrast, the TT mutant genotype was associated with higher *GAS5* levels in MS patients compared to other genotypes [29]. Furthermore, this variant has been shown to influence *GAS5* secondary structure, transcript stability, and miRNA binding sites [50].

Additionally, our functional analysis demonstrated that rs2067079 is a strong cis-eQTL regulating multiple genes beyond *GAS5*. This variant negatively impacts *DARS2* and *GAS5*-*AS1* expression, particularly in the brain, providing an alternative mechanism for its association with MS. While *DARS2* mutations are linked to childhood-to-adolescence-onset leukoencephalopathy affecting the brain and spinal cord, no enrichment has been found in MS patients [62]. *GAS5*-*AS1* modulates *GAS5* levels in THP1-derived macrophages [63], suggesting that rs2067079-mediated regulation of *GAS5*-*AS1* could indirectly affect *GAS5* expression, though further investigation is warranted. Notably, *GAS5* upregulation has been observed in the brain microglia and serum of MS patients [17, 29] and in the microglia of EAE mice brains [17].

Moreover, *GAS5* inhibits *miR*-*146a* in various inflammatory conditions [18, 19], potentially disrupting the *miR*-*146a/IRAK-1* negative feedback loop on NF-κB signaling and cytokine release. Functionally, rs2067079 exerts a trans-eQTL effect on *PRDX6*. Elevated *PRDX6* expression in astrocytes of EAE mice and MS patients has been shown to mitigate blood–brain barrier disruption and neuroinflammation [64]. *miR*-*146a* has been implicated in neuroinflammation [65, 66]. Its expression is elevated in peripheral blood mononuclear cells and the brain white-matter lesions of MS patients [65], and it enhances oligodendrogenesis by targeting *IRAK-1* in a rat stroke model [66]. Consistent with previous Egyptian reports [30, 31], the minor C and G alleles of *miR*-*146a* rs2910164 and rs57095329 were protective against MS development. We identified a highly protective interaction between these two SNPs against the risk of MS and its phenotypes, with a female-specific association. Since these SNPs were not in LD, their independent contributions to reducing MS susceptibility and progression appear significant.

This protective effect could be attributed to the influence of these variants on *miR*-*146a* expression and function [44–46]. Specifically, certain genotype combinations may reduce *miR*-*146a* expression. The rs57095329 G allele has been linked to decreased *miR*-*146a* expression in peripheral blood leukocytes of systemic lupus erythematosus patients compared to controls by reducing promoter-protein binding affinity and activity [46]. Furthermore, our findings indicated that rs57095329 is a strong cis-eQTL exerting a negative regulatory effect on *MIR3142HG*, the host gene of *miR*-*146a*, which may be processed into *miR*-*146a* rather than *miR*-*3142* [67].

Contrary to our results, these *miR*-*146a* polymorphisms were not associated with overall MS risk in the Chinese Han population. However, the rs2910164 variant exhibited a female-specific association, where the C allele increased RRMS risk in Chinese patients [45]. In that study, the C allele correlated with elevated expression of *miR*-*146a*, TNF-α, and IFN-γ, but not IL-1β, compared with the homozygous GG genotype [45]. These discrepancies could stem from differences in sample size, population genetics, and LD patterns of these loci across populations, which may cause diversity in associations between genetic variations and the pathogenesis of MS and other autoimmune diseases.

A previous study in Egyptians demonstrated an association between the single-locus effects of the *miR*-*146a* rs2910164 major G risk allele and its target gene, *IRAK-1* rs3027898 minor C risk allele, with the susceptibility to MS and its phenotypes [30]. This study reported significant associations of these SNPs with RRMS and SPMS risk compared to controls [30]. However, the present study did not replicate the association of rs2910164 with SPMS risk, likely due to the inclusion of a larger sample size and adjustments for confounders. Notably, our study provides a more comprehensive analysis by demonstrating the joint effects of these two SNPs.

Intriguingly, we identified a significant interaction between the two *miR*-*146a* variants and *IRAK-1* rs3027898 in association with overall MS risk, and in particular with SPMS risk, and in gender-stratified groups. This suggests a potential role in MS severity, progression, and gender-based heritability. Specifically, the combination of the risk alleles of *IRAK-1* rs3027898 (C allele) and *miR*-*146a* rs2910164 (G allele) was associated with an increased MS risk, whereas the interaction of the protective alleles of *IRAK-1* rs3027898 (A allele) and *miR*-*146a* rs57095329 (G allele) conferred protection against SPMS.

*IRAK-1* is predominantly expressed in CNS microglia, functioning as a negative regulator of the TLR4-MyD88 signaling pathway, and its role in innate immunity, inflammation, and MS has been well established [68, 69]. Additionally, genetic variations in *IRAK-1* can elevate its expression levels [47]. These findings highlight the critical role of genetic variations in the *miR*-*146a/IRAK-1* axis in modulating MS susceptibility and progression.

*miR*-*155* is a key driver of neuroinflammation, amplifying the autoimmune response and promoting demyelination through microglial activation, astrocyte polarization, *CD47* protein downregulation, and transcription factor modulation [70, 71]. The *miR*-*155* rs767649 variant has been identified as a predisposing factor for MS and its phenotypes [31], consistent with our single-locus analysis, which demonstrated that rs767649 confers an increased risk for MS and RRMS. However, other studies have reported no association between this SNP and MS risk [45, 72]. Although our study did not find a direct association between rs767649 and SPMS risk, we observed a significant interaction between rs2910164 and rs767649, contributing to SPMS susceptibility. This suggests an additive effect of the *miR*-*146a* rs2910164 variant in MS progression, a pattern previously reported in type 1 diabetes [73, 74].

Both *miR*-*146a* rs2910164 and *miR*-*155* rs767649 variants have been shown to alter the expression of their respective transcripts [44–46, 48, 49], potentially creating a pro-inflammatory environment that drives MS progression. Indeed, elevated *miR*-*155* and *miR*-*146a* expression has been consistently reported in MS patients [25, 26, 28, 35, 45, 74, 65]. The upregulation of *miR*-*146a* enhances *IRAK-1* targeting, which, in turn, increases IL-17 production in MS [75, 76]. Additionally, *miR*-*155* promotes the differentiation of Th1 and Th17 cell subsets, leading to elevated pro-inflammatory cytokine levels in both MS and EAE models [77].

Our functional analysis revealed an additional mechanism by which rs767649 may contribute to MS. This variant acts has a feature of cis-eQTL for *JAM2* in the brain and B cells, positively influencing its expression. *JAM2* is an adhesion molecule found in the tight junctions of epithelial and endothelial cells and acts as a ligand for various immune cell subsets. Notably, the absence of *JAM2* in vasculature has been shown to ameliorate EAE [78]. Furthermore, *JAM2* functions as a somatodendritic inhibitor of oligodendrocyte myelination in the spinal cord dorsal horn [79]. Collectively, these findings suggest *JAM2* as a potential therapeutic target for MS.

The interaction between *IRAK-1* rs3027898 and *miR*-*155* rs767649 represents another notable genetic association, significantly increasing the risk of overall MS and its subtypes in both male and female-stratified groups. Mechanistically, the presence of the *miR*-*155* rs767649 T allele enhances *miR*-*155* expression via increased transcriptional activity [48, 49]. Given that both *miR*-*155* and *IRAK-1* contribute to neuroinflammation, our findings suggest that the combination of these two SNPs might create a more pronounced inflammatory environment, thereby imposing a higher MS risk than either SNP alone. Additionally, our study and a previous Egyptian study demonstrated a female-specific association of rs767649 with MS susceptibility; however, its interaction with the *IRAK-1* SNP appears to extend this risk to males. Interestingly, a significant interaction was observed between *GAS5* and *miR*-*155* variants in females but not males, further expanding the understanding of gender-specific genetic backgrounds in MS.

Furthermore, our results highlight the protective effect of the CCAAA haplotype, which contains four protective alleles and is associated with an approximately sevenfold reduced risk of MS compared to controls. This finding mirrors other reports indicating that increasing high-risk or protective low-risk alleles magnifies the likelihood of developing or avoiding the disease, respectively [80, 81]. Our data suggest that these allelic combinations could broaden the scope of MS heritability. Since all the genetic variants investigated in this study have functional consequences on gene expression, structure, or function, combining their low-risk alleles could result in more significant protective effects.

Among MS patients, specific haplotypes were associated with gender and EDSS scores. The CGACT and CGAAT haplotypes were more prevalent in female and male MS patients, respectively, which might be attributed to the higher prevalence of the *IRAK-1* rs3027898 C risk allele in females. Paradoxically, the CCGCA haplotype, which contains only one risk allele (rs3027898 C allele), was associated with greater MS severity (EDSS ≥ 6). This unexpected finding warrants further investigation. Nevertheless, it underscores the role of *IRAK-1* rs3027898 in MS progression, possibly by altering *IRAK-1* levels and its associated signaling pathways.

Few studies have explored gene–gene interactions between MHC and immune-related genes to explain the complex heredity of MS [36–38]. Our study is the first to focus on the impact of epistatic interactions between SNPs in non-coding RNA genes and their targets, showing that these interactions have stronger combined effects than single SNPs and could serve as potential biomarkers for precision medicine in MS patients.

Nonetheless, we acknowledge certain limitations in our study. First, the study population is relatively homogeneous, with all samples collected from a single hospital, making selection bias unavoidable. Second, although the study had sufficient statistical power, the sample size was modest due to the low prevalence of MS in Egypt, resulting in relatively large or small ORs, wider confidence intervals, and potential overestimation of effect sizes. Third, we included only one population, which may limit the generalizability of our findings. Fourth, we did not assess the expression levels of affected transcripts in MS patients with different genotypes and/or combined genotypes from the studied population; however, prior studies have documented the functional impact of these SNPs at the individual level in other populations. Nevertheless, our findings are a critical first step toward prioritizing candidate variants for further functional validation, particularly in the Egyptian population. Fifth, we included only SNPs with higher MAF (> 10%). This approach may exclude rare but biologically functional variants; however, it preserves statistical power and ensures that the analyzed SNPs are informative and relevant to the genetic background of the population while minimizing noise from very rare variants.

Despite these limitations, this study is a preliminary investigation, warranting further replication in independent cohorts, including those of different ethnic and geographic origins. Future multicenter studies with larger sample sizes will be essential to validate these interactions and assess their generalizability across diverse populations.

## Conclusion

We propose that the magnified combined effect of SNPs in *GAS5*, *miR*-*146a*, *IRAK-1*, and *miR*-*155a* genes may influence the risk of MS and its phenotypes through epistatic interactions and could aid in the risk stratification of MS patients. These findings expand the understanding of the genetic complexity of MS and may be leveraged in genetic counseling to facilitate early diagnosis, monitoring, and optimal treatment of MS patients.

## Supplementary Information

Below is the link to the electronic supplementary material.

Supplementary file1 (DOCX 1174 KB)

## Data Availability

Data is provided within the manuscript or supplementary information files. While individual-level data are not publicly available, they can be provided upon reasonable request for research purposes.
