# Supplementary material for: Association of Epistatic Effects of lncRNA GAS5, miR-146a, IRAK-1, and miR-155 Genetic Variants with Multiple Sclerosis Risk and Severity
Source: Mol Neurobiol. 2025 Apr 15;62(8):10742–64. doi: 10.1007/s12035-025-04876-8 (PMC12289807; doi:10.1007/s12035-025-04876-8)
Supplement: Supplementary file 1 — Supplementary file1 (DOCX 1174 KB) [file 12035_2025_4876_MOESM1_ESM.docx]

**Supplementary Information**

**Table S1. Hardy-Weinberg equilibrium of studied SNPs in the study groups**

| **GAS5 rs2067079 (C/T)** | | | | | | |
| --- | --- | --- | --- | --- | --- | --- |
|  | **CC** | **CT** | **TT** | **C** | **T** | ***P*-value** |
| **Control** | 60 | 56 | 4 | 176 | 64 | 0.059 |
| **MS** | 58 | 42 | 16 | 158 | 74 | 0.086 |
| **miR-146a rs2910164 (C/G)** | | | | | | |
|  | **GG** | **GC** | **CC** | **G** | **C** | ***P*-value** |
| **Control** | 32 | 68 | 20 | 132 | 108 | 0.14 |
| **MS** | 41 | 62 | 13 | 144 | 88 | 0.17 |
| **miR-146a rs57095329 (A/G)** | | | | | | |
|  | **AA** | **AG** | **GG** | **A** | **G** | ***P*-value** |
| **Control** | 30 | 70 | 20 | 130 | 110 | 0.068 |
| **MS** | 81 | 31 | 4 | 193 | 39 | 0.74 |
| **IRAK-1 rs3027898 (A/C)** | | | | | | |
|  | **AA** | **AC** | **CC** | **A** | **C** | ***P*-value** |
| **Control** | 80 | 32 | 8 | 192 | 48 | 0.083 |
| **MS** | 45 | 48 | 23 | 138 | 94 | 0.13 |
| **miR-155 rs767649 (A/T)** | | | | | | |
|  | **AA** | **AT** | **TT** | **A** | **T** | ***P*-value** |
| **Control** | 47 | 63 | 10 | 157 | 83 | 0.11 |
| **MS** | 14 | 63 | 39 | 91 | 141 | 0.17 |

The exact test was used for HWE calculations. The analysis was performed using SNPStats online software.

**Table S2. Association of GAS5 rs2067079 (C/T) polymorphism with the risk of MS**

| **GAS5 rs2067079 (C/T)** | | | | | | | |
| --- | --- | --- | --- | --- | --- | --- | --- |
| **Model** | **Genotype**  **/allele** | **Control**  **(n=120)** | **MS**  **(n=116)** | **OR (95% CI)** | ***P^a^*-value** | **AIC** | **BIC** |
| Codominant | CC | 60 (50%) | 58 (50%) | 1.00 | **0.013** | 324.4 | 345.2 |
|  | CT | 56 (46.7%) | 42 (36.2%) | 0.88 (0.50-1.53) |  |  |  |
|  | TT | 4 (3.3%) | 16 (13.8%) | **4.34 (1.35-13.97)** |  |  |  |
| Dominant | CC | 60 (50%) | 58 (50%) | 1.00 | 0.67 | 331 | 348.3 |
|  | CT-TT | 60 (50%) | 58 (50%) | 1.12 (0.66-1.91) |  |  |  |
| Recessive* | CC-CT | 116 (96.7%) | 100 (86.2%) | 1.00 | **0.0035** | 322.7 | 340 |
|  | TT | 4 (3.3%) | 16 (13.8%) | **4.62 (1.48-14.43)** |  |  |  |
| Overdominant | CC-TT | 64 (53.3%) | 74 (63.8%) | 1.00 | 0.23 | 329.7 | 347.1 |
|  | CT | 56 (46.7%) | 42 (36.2%) | 0.72 (0.42-1.23) |  |  |  |
| Log-additive | --- | --- | --- | 1.40 (0.92-2.12) | 0.11 | 328.7 | 346 |

* Represents the best fit model based on the lowest Akaike Information Criterion (AIC) and Bayesian Information Criterion (BIC). ^a^ adjusted for age and sex in a logistic regression model. Bold indicates statistical significance, *P*<0.05.

**Table S3. Association of miR-146a rs2910164 (C/G) polymorphism with the risk of MS**

| **miR-146a rs2910164 (C/G)** | | | | | | | |
| --- | --- | --- | --- | --- | --- | --- | --- |
| **Model** | **Genotype**  **/allele** | **Control**  **(n=120)** | **MS**  **(n=116)** | **OR (95% CI)** | ***P^a^*-value** | **AIC** | **BIC** |
| Codominant | GG | 32 (26.7%) | 41 (35.3%) | 1.00 | 0.072 | 327.9 | 348.7 |
|  | CG | 68 (56.7%) | 62 (53.5%) | 0.58 (0.31-1.08) |  |  |  |
|  | CC | 20 (16.7%) | 13 (11.2%) | **0.39 (0.16-0.95)** |  |  |  |
| Dominant | GG | 32 (26.7%) | 41 (35.3%) | 1.00 | 0.036 | 326.8 | 344.1 |
|  | CG-CC | 88 (73.3%) | 75 (64.7%) | **0.54 (0.30-0.97)** |  |  |  |
| Recessive | GG-CG | 100 (83.3%) | 103 (88.8%) | 1.00 | 0.13 | 328.9 | 346.2 |
|  | CC | 20 (16.7%) | 13 (11.2%) | 0.55 (0.25-1.21) |  |  |  |
| Overdominant | GG-CC | 52 (43.3%) | 54 (46.5%) | 1.00 | 0.37 | 330.4 | 347.7 |
|  | CG | 68 (56.7%) | 62 (53.5%) | 0.78 (0.45-1.34) |  |  |  |
| Log-additive* | --- | --- | --- | **0.62 (0.40-0.94)** | **0.022** | 325.9 | 343.3 |

*****Represents the best fit model based on the lowest Akaike Information Criterion (AIC) and Bayesian Information Criterion (BIC). ^a^ adjusted for age and sex in a logistic regression model. Bold indicates statistical significance, *P*<0.05.

**Table S4. Association of miR-146a rs57095329 (A/G) polymorphism with the risk of MS**

| **miR-146a rs57095329 (A/G)** | | | | | | | |
| --- | --- | --- | --- | --- | --- | --- | --- |
| **Model** | **Genotype**  **/allele** | **Control**  **(n=120)** | **MS**  **(n=116)** | **OR (95% CI)** | ***P^a^*-value** | **AIC** | **BIC** |
| Codominant | AA | 30 (25%) | 81 (69.8%) | 1.00 | <0.0001 | 282.8 | 303.6 |
|  | AG | 70 (58.3%) | 31 (26.7%) | **0.17 (0.09-0.30)** |  |  |  |
|  | GG | 20 (16.7%) | 4 (3.5%) | **0.06 (0.02-0.23)** |  |  |  |
| Dominant | AA | 30 (25%) | 81 (69.8%) | 1.00 | <0.0001 | 283.5 | 300.8 |
|  | AG-GG | 90 (75%) | 35 (30.2%) | **0.14 (0.08-0.26)** |  |  |  |
| Recessive | AA-AG | 100 (83.3%) | 112 (96.5%) | 1.00 | 5e-04 | 319 | 336.3 |
|  | GG | 20 (16.7%) | 4 (3.5%) | **0.15 (0.04-0.53)** |  |  |  |
| Overdominant | AA-GG | 50 (41.7%) | 85 (73.3%) | 1.00 | <0.0001 | 306.8 | 324.2 |
|  | AG | 70 (58.3%) | 31 (26.7%) | **0.26 (0.15-0.45)** |  |  |  |
| Log-additive* | --- | --- | --- | **0.19 (0.12-0.32)** | <0.0001 | 281.8 | 299.1 |

*****Represents the best fit model based on the lowest Akaike Information Criterion (AIC) and Bayesian Information Criterion (BIC). ^a^ adjusted for age and sex in a logistic regression model. Bold indicates statistical significance, *P*<0.05.

**Table S5. Association of IRAK-1 rs3027898 (A/C) polymorphism with the risk of MS**

| **IRAK-1 rs3027898 (A/C)** | | | | | | | |
| --- | --- | --- | --- | --- | --- | --- | --- |
| **Model** | **Genotype**  **/allele** | **Control**  **(n=120)** | **MS**  **(n=116)** | **OR (95% CI)** | ***P^a^*-value** | **AIC** | **BIC** |
| Codominant | AA | 80 (66.7%) | 45 (38.8%) | 1.00 | 2e-04 | 316.3 | 337.1 |
|  | AC | 32 (26.7%) | 48 (41.4%) | **2.36 (1.29-4.34)** |  |  |  |
|  | CC | 8 (6.7%) | 23 (19.8%) | **4.82 (1.97-11.78)** |  |  |  |
| Dominant | AA | 80 (66.7%) | 45 (38.8%) | 1.00 | 1e-04 | 316.6 | 333.9 |
|  | AC-CC | 40 (33.3%) | 71 (61.2%) | **2.89 (1.66-5.03)** |  |  |  |
| Recessive | AA-AC | 112 (93.3%) | 93 (80.2%) | 1.00 | 0.0026 | 322.1 | 339.4 |
|  | CC | 8 (6.7%) | 23 (19.8%) | **3.56 (1.48-8.55)** |  |  |  |
| Overdominant | AA-CC | 88 (73.3%) | 68 (58.6%) | 1.00 | 0.061 | 327.7 | 345 |
|  | AC | 32 (26.7%) | 48 (41.4%) | 1.73 (0.97-3.08) |  |  |  |
| Log-additive* | --- | --- | --- | **2.25 (1.50-3.35)** | <0.0001 | 314.3 | 331.6 |

*****Represents the best fit model based on the lowest Akaike Information Criterion (AIC) and Bayesian Information Criterion (BIC). ^a^ adjusted for age and sex in a logistic regression model. Bold indicates statistical significance, *P*<0.05.

**Table S6. Association of miR-155 rs767649 (A/T) polymorphism with the risk of MS**

| **miR-155 rs767649 (A/T)** | | | | | | | |
| --- | --- | --- | --- | --- | --- | --- | --- |
| **Model** | **Genotype**  **/allele** | **Control**  **(n=120)** | **MS**  **(n=114)** | **OR (95% CI)** | ***P^a^*-value** | **AIC** | **BIC** |
| Codominant | AA | 47 (39.2%) | 14 (12.1%) | 1.00 | <0.0001 | 297.3 | 318.1 |
|  | AT | 63 (52.5%) | 63 (54.3%) | **3.61 (1.76-7.41)** |  |  |  |
|  | TT | 10 (8.3%) | 39 (33.6%) | **13.22 (5.19-33.67)** |  |  |  |
| Dominant | AA | 47 (39.2%) | 14 (12.1%) | 1.00 | <0.0001 | 307.2 | 324.5 |
|  | AT-TT | 73 (60.8%) | 102 (87.9%) | **5.01 (2.50-10.05)** |  |  |  |
| Recessive | AA-AT | 110 (91.7%) | 77 (66.4%) | 1.00 | <0.0001 | 309.1 | 326.4 |
|  | TT | 10 (8.3%) | 39 (33.6%) | **5.32 (2.49-11.38)** |  |  |  |
| Overdominant | AA-TT | 57 (47.5%) | 53 (45.7%) | 1.00 | 0.67 | 331 | 348.3 |
|  | AT | 63 (52.5%) | 63 (54.3%) | 1.12 (0.66-1.90) |  |  |  |
| Log-additive* | --- | --- | --- | **3.64 (2.28-5.79)** | <0.0001 | 295.3 | 312.7 |

*Represents the best fit model based on the lowest Akaike Information Criterion (AIC) and Bayesian Information Criterion (BIC). ^a^ adjusted for age and sex in a logistic regression model. Bold indicates statistical significance, *P*<0.05.

**Table S7. Cross-interaction of individual SNPs with gender as a risk factor for MS using the codominant model**

| **GAS5 rs2067079 and Sex cross-classification interaction** | | | | | | |
| --- | --- | --- | --- | --- | --- | --- |
|  | **Control**  **(n=40)** | **MS**  **(n=32)** | **Adjusted OR^a^**  **(95% CI)** | **Control**  **(n=80)** | **MS**  **(n=84)** | **Adjusted OR^a^ (95% CI)** |
| **Genotype** | **Males** | | | **Females** | | |
| **CC** | 13 | 12 | 1.00 | 47 | 46 | 1.13 (0.46-2.75) |
| **CT** | 24 | 15 | 0.72 (0.26-2.01) | 32 | 27 | 1.02 (0.39-2.65) |
| **TT** | 3 | 5 | 1.71 (0.33-8.81) | 1 | 11 | 12.62 (1.39-114.42) |
| **Interaction *P*^a^-value:**0.34 | | | | | | |
| **miR-146a rs2910164 and Sex cross-classification interaction** | | | | | | |
| **GG** | 8 | 16 | 1.00 | 24 | 25 | 0.62 (0.22-1.75) |
| **CG** | 32 | 12 | **0.17 (0.06-0.50)** | 36 | 50 | 0.64 (0.24-1.67) |
| **CC** | 0 | 4 | --- | 20 | 9 | **0.23 (0.07-0.73)** |
| **Interaction *P*^a^-value: <0.0001** | | | | | | |
| **miR-146a rs57095329 and Sex cross-classification interaction** | | | | | | |
| **AA** | 7 | 22 | 1.00 | 23 | 59 | 0.86 (0.32-2.31) |
| **AG** | 23 | 9 | 0.13 (0.04-0.40) | 47 | 22 | 0.16 (0.06-0.43) |
| **GG** | 10 | 1 | 0.04 (0.00-0.34) | 10 | 3 | 0.11 (0.02-0.50) |
| **Interaction *P*^a^-value:**0.61 | | | | | | |
| **IRAK-1 rs3027898 and Sex cross-classification interaction** | | | | | | |
| **AA** | 26 | 17 | 1.00 | 54 | 28 | 0.84 (0.39-1.82) |
| **AC** | 10 | 9 | 1.21 (0.39-3.72) | 22 | 39 | 2.64 (1.18-5.92) |
| **CC** | 4 | 6 | 2.63 (0.62-11.11) | 4 | 17 | 6.21 (1.77-21.76) |
| **Interaction *P*^a^-value:**0.26 | | | | | | |
| **miR-155 rs767649 and Sex cross-classification interaction** | | | | | | |
| **AA** | 12 | 3 | 1.00 | 35 | 11 | 1.25 (0.30-5.27) |
| **AT** | 28 | 19 | 2.53 (0.62-10.27) | 35 | 44 | **4.88 (1.27-18.73)** |
| **TT** | 0 | 10 | --- | 10 | 29 | **10.98 (2.55-47.32)** |
| **Interaction *P*^a^-value: 0.027** | | | | | | |

Data were computed using the SNPStats online software using the codominant model. ^a^ adjusted with age in a logistic regression model. *P*<0.05 (bold) is statistically significant. CI, confidence interval; CRC, colorectal cancer; OR, odds ratio.

**Table S8. Association of gene-gene interaction of SNPs with MS risk in females**

|  | **Female Control** | | | **FemaleMS** | **Adjusted OR (95% CI)** | **Female Control** | | | | | | | | **FemaleMS** | | | | **Adjusted OR (95% CI)** | **Female**  **Control** | | | | | | | | **Female**  **MS** | | | | | | | | | **Adjusted OR (95% CI)** | | |
| --- | --- | --- | --- | --- | --- | --- | --- | --- | --- | --- | --- | --- | --- | --- | --- | --- | --- | --- | --- | --- | --- | --- | --- | --- | --- | --- | --- | --- | --- | --- | --- | --- | --- | --- | --- | --- | --- | --- |
| **GAS5 rs2067079 and miR-146a rs2910164 cross-classification interaction** | | | | | | | | | | | | | | | | | | | | | | | | | | | | | | | | | | | | | | |
|  | **GG** | | | | | | **CG** | | | | | | | | | | | | **CC** | | | | | | | | | | | | | | | | | | | |
| **CC** | 12 | | 14 | | 1.00 | | 19 | | | | | | | 28 | | | | 1.09 (0.40-2.94) | 16 | | | | | | | | 4 | | | | | | | | | 0.19 (0.05-0.73) | | |
| **CT** | 12 | | 5 | | 0.46 (0.12-1.74) | | 16 | | | | | | | 18 | | | | 0.80 (0.28-2.30) | 4 | | | | | | | | 4 | | | | | | | | | 0.95 (0.19-4.76) | | |
| **TT** | 0 | | 6 | | --- | | 1 | | | | | | | 4 | | | | 2.22 (0.21-23.91) | 0 | | | | | | | | 1 | | | | | | | | | --- | | |
| **Interaction p-value:**0.095 | | | | | | | | | | | | | | | | | | | | | | | | | | | | | | | | | | | | | | |
| **GAS5 rs2067079 and miR-146a rs57095329 cross-classification interaction** | | | | | | | | | | | | | | | | | | | | | | | | | | | | | | | | | | | | | | |
|  | | **AA** | | | | | | **AG** | | | | | | | | | | | **GG** | | | | | | | | | | | | | | | | | | | |
| **CC** | | 20 | 32 | | 1.00 | | | 20 | | | | | | 12 | | | | **0.38**  **(0.15-0.94)** | 7 | | | | | | | | 2 | | | | | | | | | 0.20 (0.04-1.10) | | |
| **CT** | | 2 | 19 | | **7.17**  **(1.45-35.45)** | | | 27 | | | | | | 8 | | | | **0.19**  **(0.07-0.52)** | 3 | | | | | | | | 0 | | | | | | | | | 0.00 | | |
| **TT** | | 1 | 8 | | 5.03 (0.57-44.55) | | | 0 | | | | | | 2 | | | | --- | 0 | | | | | | | | 1 | | | | | | | | | --- | | |
| **Interaction p-value: 0.014** | | | | | | | | | | | | | | | | | | | | | | | | | | | | | | | | | | | | | | |
| **GAS5 rs2067079 and IRAK-1 rs3027898 cross-classification interaction** | | | | | | | | | | | | | | | | | | | | | | | | | | | | | | | | | | | | | | |
|  | | **AA** | | | | | | **AC** | | | | | | | | | | | | **CC** | | | | | | | | | | | | | | | | | | |
| **CC** | | 33 | 14 | | 1.00 | | | 10 | | | | | | 23 | | | | **4.96**  **(1.84-13.34)** | | 4 | | | | | | 9 | | | | | | | **4.77**  **(1.23-18.48)** | | | | | |
| **CT** | | 21 | 11 | | 1.30 (0.49-3.43) | | | 11 | | | | | | 11 | | | | 2.18 (0.75-6.28) | | 0 | | | | | | 5 | | | | | | | --- | | | | | |
| **TT** | | 0 | 3 | | --- | | | 1 | | | | | | 5 | | | | **11.40**  **(1.21-107.43)** | | 0 | | | | | | 3 | | | | | | | --- | | | | | |
| **Interaction p-value:**0.1 | | | | | | | | | | | | | | | | | | | | | | | | | | | | | | | | | | | | | | |
| **GAS5 rs2067079 and miR-155 rs767649 cross-classification interaction table** | | | | | | | | | | | | | | | | | | | | | | | | | | | | | | | | | | | | | | |
|  | | **AA** | | | | | | **AT** | | | | | | | | | | | | | **TT** | | | | | | | | | | | | | | | | | |
| **CC** | | 12 | 8 | | 1.00 | | | 28 | | | | | | 22 | | 1.22 (0.42-3.52) | | | | | 7 | | | | 16 | | | | | | | 3.47 (0.98-12.33) | | | | | | |
| **CT** | | 23 | 3 | | 0.22 (0.05-1.00) | | | 7 | | | | | | 15 | | 3.43 (0.96-12.30) | | | | | 2 | | | | 9 | | | | | | | **6.71 (1.12-40.01)** | | | | | | |
| **TT** | | 0 | 0 | | --- | | | 0 | | | | | | 7 | | --- | | | | | 1 | | | | 4 | | | | | | | 6.00 (0.55-66.00) | | | | | | |
| **Interaction p-value: 0.01** | | | | | | | | | | | | | | | | | | | | | | | | | | | | | | | | | | | | | | |
| **miR-146a rs2910164 and miR-146a rs57095329 cross-classification interaction** | | | | | | | | | | | | | | | | | | | | | | | | | | | | | | | | | | | | | | |
|  | | **AA** | | | | | | | **AG** | | | | | | | | | | | | | **GG** | | | | | | | | | | | | | | | | |
| **GG** | | 1 | 22 | | 1.00 | | | | 20 | | | | | 1 | | **0.00 (0.00-0.04)** | | | | | | 3 | | | | | 2 | | | | | | | | | **0.03**  **(0.00-0.47)** | | |
| **CG** | | 14 | 30 | | **0.08 (0.01-0.71)** | | | | 15 | | | | | 20 | | **0.05 (0.01-0.44)** | | | | | | 7 | | | | | 0 | | | | | | | | | 0.00 | | |
| **CC** | | 8 | 7 | | **0.04**  **(0.00-0.35)** | | | | 12 | | | | | 1 | | **0.00**  **(0.00-0.06)** | | | | | | 0 | | | | | 1 | | | | | | | | | --- | | |
| **Interaction p-value: <0.0001** | | | | | | | | | | | | | | | | | | | | | | | | | | | | | | | | | | | | | | |
| **miR-146a rs2910164 and IRAK-1 rs3027898 cross-classification interaction** | | | | | | | | | | | | | | | | | | | | | | | | | | | | | | | | | | | | | | |
|  | | **AA** | | | | | | | **AC** | | | | | | | | | | | | | **CC** | | | | | | | | | | | | | | | | |
| **GG** | | 19 | 4 | | 1.00 | | | | 5 | | | | | 14 | | | **12.81**  **(2.77-59.28)** | | | | | 0 | | | | | | 7 | | | | | | | | --- | | |
| **CG** | | 19 | 18 | | **4.29**  **(1.12-16.47)** | | | | 13 | | | | | 25 | | | **8.54**  **(2.01-36.21)** | | | | | 4 | | | | | | 7 | | | | | | | | **7.80**  **(1.35-45.23)** | | |
| **CC** | | 16 | 6 | | 1.71  (0.38-7.60) | | | | 4 | | | | | 0 | | | 0.00 | | | | | 0 | | | | | | 3 | | | | | | | | --- | | |
| **Interaction p-value: 0.00097** | | | | | | | | | | | | | | | | | | | | | | | | | | | | | | | | | | | | | | |
| **miR-146a rs2910164 and miR-155 rs767649 cross-classification interaction** | | | | | | | | | | | | | | | | | | | | | | | | | | | | | | | | | | | | | | |
|  | | **AA** | | | | | | | | **AT** | | | | | | | | | | | | | **TT** | | | | | | | | | | | | | | | |
| **GG** | | 8 | 1 | | 1.00 | | | | | 12 | | | | | 17 | | 8.32 (0.85-81.66) | | | | | | 4 | | | | | | | | 7 | | | | | | | 11.38  (0.98-131.65) |
| **CG** | | 15 | 8 | | 2.80  (0.26-30.60) | | | | | 15 | | | | | 22 | | 8.34 (0.86-81.11) | | | | | | 6 | | | | | | | | 20 | | | | | | | **17.47**  **(1.57-193.82)** |
| **CC** | | 12 | 2 | | 1.01  (0.07-13.84) | | | | | 8 | | | | | 5 | | 3.51 (0.30-41.05) | | | | | | 0 | | | | | | | | 2 | | | | | | | --- |
| **Interaction p-value:**0.61 | | | | | | | | | | | | | | | | | | | | | | | | | | | | | | | | | | | | | | |
| **miR-146a rs57095329 and IRAK-1 rs3027898 cross-classification interaction** | | | | | | | | | | | | | | | | | | | | | | | | | | | | | | | | | | | | | | |
|  | | **AA** | | | | | | | | | **AC** | | | | | | | | | | | | **CC** | | | | | | | | | | | | | | | |
| **AA** | | 15 | 24 | | 1.00 | | | | | | 4 | | | 22 | | | 3.45 (0.99-12.01) | | | | | | 4 | | | | | | | 13 | | | | | | | 2.10 (0.57-7.71) | |
| **AG** | | 33 | 4 | | **0.07**  **(0.02-0.25)** | | | | | | 14 | | | 17 | | | 0.80 (0.30-2.14) | | | | | | 0 | | | | | | | 1 | | | | | | | --- | |
| **GG** | | 6 | 0 | | 0.00 | | | | | | 4 | | | 0 | | | 0.00 | | | | | | 0 | | | | | | | 3 | | | | | | | --- | |
| **Interaction p-value: 0.0042** | | | | | | | | | | | | | | | | | | | | | | | | | | | | | | | | | | | | | | |
| **miR-146a rs57095329 and miR-155 rs767649 cross-classification interaction** | | | | | | | | | | | | | | | | | | | | | | | | | | | | | | | | | | | | | | |
|  | | **AA** | | | | | | | | | | **AT** | | | | | | | | | | | **TT** | | | | | | | | | | | | | | | |
| **AA** | | 0 | 2 | | 1.00 | | | | | | | 17 | | 38 | | | | 0.00 | | | | | 6 | | | | | | 19 | | | | | 0.00 | | | | |
| **AG** | | 28 | 8 | | 0.00 | | | | | | | 15 | | 4 | | | | 0.00 | | | | | 4 | | | | | | 10 | | | | | 0.00 | | | | |
| **GG** | | 7 | 1 | | 0.00 | | | | | | | 3 | | 2 | | | | 0.00 | | | | | 0 | | | | | | 0 | | | | | --- | | | | |
| **Interaction p-value:**0.056 | | | | | | | | | | | | | | | | | | | | | | | | | | | | | | | | | | | | | | |
| **IRAK-1 rs3027898 and miR-155 rs767649 cross-classification interaction** | | | | | | | | | | | | | | | | | | | | | | | | | | | | | | | | | | | | | | |
|  | | **AA** | | | | | | | | | | | **AT** | | | | | | | | | | | **TT** | | | | | | | | | | | | | | |
| **AA** | | 22 | 0 | | 1.00 | | | | | | | | 25 | 22 | | | | --- | | | | | | 7 | | | | | | 6 | | | | | --- | | | |
| **AC** | | 13 | 8 | | --- | | | | | | | | 6 | 18 | | | | --- | | | | | | 3 | | | | | | 13 | | | | | --- | | | |
| **CC** | | 0 | 3 | | --- | | | | | | | | 4 | 4 | | | | --- | | | | | | 0 | | | | | | 10 | | | | | --- | | | |
| **Interaction p-value: 0.0014** | | | | | | | | | | | | | | | | | | | | | | | | | | | | | | | | | | | | | | |
|  | | | | | | | | | | | | | | | | | | | | | | | | | | | | | | | | | | | | | | |

The association analysis was performed using SNPStats online software. All interactions were performed in the codominant model (control, n=80 vs. MS, n=84). ^a^ adjusted for age in a logistic regression model. OR and *P*-values in bold are statistically significant, *P* < 0.05.

**Table S9. Association of gene-gene interaction of SNPs with MS risk in males**

|  | **Male Control** | | | **Male**  **MS** | **Adjusted OR (95% CI)** | **Male Control** | | | | | | | | **Male**  **MS** | | | | **Adjusted OR (95% CI)** | **Male Control** | | | | | | | | **Male**  **MS** | | | | | | | | | **Adjusted OR (95% CI)** | | |
| --- | --- | --- | --- | --- | --- | --- | --- | --- | --- | --- | --- | --- | --- | --- | --- | --- | --- | --- | --- | --- | --- | --- | --- | --- | --- | --- | --- | --- | --- | --- | --- | --- | --- | --- | --- | --- | --- | --- |
| **GAS5 rs2067079 and miR-146a rs2910164 cross-classification interaction** | | | | | | | | | | | | | | | | | | | | | | | | | | | | | | | | | | | | | | |
|  | **GG** | | | | | | **CG** | | | | | | | | | | | | **CC** | | | | | | | | | | | | | | | | | | | |
| **CC** | 1 | | 6 | | 1.00 | | 12 | | | | | | | 6 | | | | **0.09 (0.01-0.93)** | 0 | | | | | | | | 0 | | | | | | | | | --- | | |
| **CT** | 4 | | 8 | | 0.47 (0.04-5.86) | | 20 | | | | | | | 4 | | | | **0.03 (0.00-0.37)** | 0 | | | | | | | | 3 | | | | | | | | | --- | | |
| **TT** | 3 | | 2 | | 0.11 (0.01-1.79) | | 0 | | | | | | | 2 | | | | --- | 0 | | | | | | | | 1 | | | | | | | | | --- | | |
| **Interaction p-value: 0.039** | | | | | | | | | | | | | | | | | | | | | | | | | | | | | | | | | | | | | | |
| **GAS5 rs2067079 and miR-146a rs57095329 cross-classification interaction** | | | | | | | | | | | | | | | | | | | | | | | | | | | | | | | | | | | | | | |
|  | | **AA** | | | | | | **AG** | | | | | | | | | | | **GG** | | | | | | | | | | | | | | | | | | | |
| **CC** | | 2 | 8 | | 1.00 | | | 8 | | | | | | 4 | | | | **0.10 (0.01-0.78)** | 3 | | | | | | | | 0 | | | | | | | | | 0.00 | | |
| **CT** | | 5 | 10 | | 0.43 (0.06-2.93) | | | 12 | | | | | | 4 | | | | **0.07 (0.01-0.50)** | 7 | | | | | | | | 1 | | | | | | | | | **0.02 (0.00-0.34)** | | |
| **TT** | | 0 | 4 | | --- | | | 3 | | | | | | 1 | | | | 0.08 (0.01-1.25) | 0 | | | | | | | | 0 | | | | | | | | | --- | | |
| **Interaction p-value:**0.43 | | | | | | | | | | | | | | | | | | | | | | | | | | | | | | | | | | | | | | |
| **GAS5 rs2067079 and IRAK-1 rs3027898 cross-classification interaction** | | | | | | | | | | | | | | | | | | | | | | | | | | | | | | | | | | | | | | |
|  | | **AA** | | | | | | **AC** | | | | | | | | | | | | **CC** | | | | | | | | | | | | | | | | | | |
| **CC** | | 11 | 7 | | 1.00 | | | 2 | | | | | | 4 | | | | 3.20 (0.40-25.62) | | 0 | | | | | | 1 | | | | | | | --- | | | | | |
| **CT** | | 15 | 7 | | 0.73 (0.20-2.71) | | | 5 | | | | | | 3 | | | | 0.96 (0.15-6.06) | | 4 | | | | | | 5 | | | | | | | 1.93 (0.34-11.07) | | | | | |
| **TT** | | 0 | 3 | | --- | | | 3 | | | | | | 2 | | | | 1.06 (0.14-8.27) | | 0 | | | | | | 0 | | | | | | | --- | | | | | |
| **Interaction p-value:**0.12 | | | | | | | | | | | | | | | | | | | | | | | | | | | | | | | | | | | | | | |
| **GAS5 rs2067079 and miR-155 rs767649 cross-classification interaction table** | | | | | | | | | | | | | | | | | | | | | | | | | | | | | | | | | | | | | | |
|  | | **AA** | | | | | | **AT** | | | | | | | | | | | | | **TT** | | | | | | | | | | | | | | | | | |
| **CC** | | 4 | 1 | | 1.00 | | | 9 | | | | | | 6 | | 3.66 (0.28-47.92) | | | | | 0 | | | | 5 | | | | | | | --- | | | | | | |
| **CT** | | 8 | 2 | | 1.18 (0.08-18.15) | | | 16 | | | | | | 10 | | 3.08 (0.28-34.37) | | | | | 0 | | | | 3 | | | | | | | --- | | | | | | |
| **TT** | | 0 | 0 | | --- | | | 3 | | | | | | 3 | | 5.44 (0.32-93.27) | | | | | 0 | | | | 2 | | | | | | | --- | | | | | | |
| **Interaction p-value:**1 | | | | | | | | | | | | | | | | | | | | | | | | | | | | | | | | | | | | | | |
| **miR-146a rs2910164 and miR-146a rs57095329 cross-classification interaction** | | | | | | | | | | | | | | | | | | | | | | | | | | | | | | | | | | | | | | |
|  | | **AA** | | | | | | | **AG** | | | | | | | | | | | | | **GG** | | | | | | | | | | | | | | | | |
| **GG** | | 1 | 12 | | 1.00 | | | | 3 | | | | | 4 | | 0.10 (0.01-1.30) | | | | | | 4 | | | | | 0 | | | | | | | | | 0.00 | | |
| **CG** | | 6 | 9 | | 0.11 (0.01-1.13) | | | | 20 | | | | | 3 | | **0.01 (0.00-0.12)** | | | | | | 6 | | | | | 0 | | | | | | | | | 0.00 | | |
| **CC** | | 0 | 1 | | --- | | | | 0 | | | | | 2 | | --- | | | | | | 0 | | | | | 1 | | | | | | | | | --- | | |
| **Interaction p-value:**1 | | | | | | | | | | | | | | | | | | | | | | | | | | | | | | | | | | | | | | |
| **miR-146a rs2910164 and IRAK-1 rs3027898 cross-classification interaction** | | | | | | | | | | | | | | | | | | | | | | | | | | | | | | | | | | | | | | |
|  | | **AA** | | | | | | | **AC** | | | | | | | | | | | | | **CC** | | | | | | | | | | | | | | | | |
| **GG** | | 0 | 10 | | 1.00 | | | | 4 | | | | | 4 | | | 0.00 | | | | | 4 | | | | | | 2 | | | | | | | | 0.00 | | |
| **CG** | | 26 | 6 | | 0.00 | | | | 6 | | | | | 5 | | | 0.00 | | | | | 0 | | | | | | 1 | | | | | | | | 1.06 | | |
| **CC** | | 0 | 1 | | 1.22 | | | | 0 | | | | | 0 | | | --- | | | | | 0 | | | | | | 3 | | | | | | | | 0.85 | | |
| **Interaction p-value: 0.0012** | | | | | | | | | | | | | | | | | | | | | | | | | | | | | | | | | | | | | | |
| **miR-146a rs2910164 and miR-155 rs767649 cross-classification interaction** | | | | | | | | | | | | | | | | | | | | | | | | | | | | | | | | | | | | | | |
|  | | **AA** | | | | | | | | **AT** | | | | | | | | | | | | | **TT** | | | | | | | | | | | | | | | |
| **GG** | | 0 | 0 | | 1.00 | | | | | 8 | | | | | 12 | | 0.00 | | | | | | 0 | | | | | | | | 4 | | | | | | | 1.00 |
| **CG** | | 12 | 1 | | 0.00 | | | | | 20 | | | | | 5 | | 0.00 | | | | | | 0 | | | | | | | | 6 | | | | | | | --- |
| **CC** | | 0 | 2 | | 1.13 | | | | | 0 | | | | | 2 | | 1.04 | | | | | | 0 | | | | | | | | 0 | | | | | | | --- |
| **Interaction p-value:**1 | | | | | | | | | | | | | | | | | | | | | | | | | | | | | | | | | | | | | | |
| **miR-146a rs57095329 and IRAK-1 rs3027898 cross-classification interaction** | | | | | | | | | | | | | | | | | | | | | | | | | | | | | | | | | | | | | | |
|  | | **AA** | | | | | | | | | **AC** | | | | | | | | | | | | **CC** | | | | | | | | | | | | | | | |
| **AA** | | 5 | 12 | | 1.00 | | | | | | 2 | | | 8 | | | 1.96 (0.26-14.70) | | | | | | 0 | | | | | | | 2 | | | | | | | --- | |
| **AG** | | 15 | 5 | | **0.14 (0.03-0.59)** | | | | | | 8 | | | 1 | | | **0.06 (0.01-0.68)** | | | | | | 0 | | | | | | | 3 | | | | | | | --- | |
| **GG** | | 6 | 0 | | 0.00 | | | | | | 0 | | | 0 | | | --- | | | | | | 4 | | | | | | | 1 | | | | | | | 0.08 (0.01-1.24) | |
| **Interaction p-value:**0.78 | | | | | | | | | | | | | | | | | | | | | | | | | | | | | | | | | | | | | | |
| **miR-146a rs57095329 and miR-155 rs767649 cross-classification interaction** | | | | | | | | | | | | | | | | | | | | | | | | | | | | | | | | | | | | | | |
|  | | **AA** | | | | | | | | | | **AT** | | | | | | | | | | | **TT** | | | | | | | | | | | | | | | |
| **AA** | | 0 | 0 | | 1.00 | | | | | | | 7 | | 16 | | | | 0.00 | | | | | 0 | | | | | | 6 | | | | | 1.62 | | | | |
| **AG** | | 12 | 3 | | 0.00 | | | | | | | 11 | | 2 | | | | 0.00 | | | | | 0 | | | | | | 4 | | | | | --- | | | | |
| **GG** | | 0 | 0 | | --- | | | | | | | 10 | | 1 | | | | 0.00 | | | | | 0 | | | | | | 0 | | | | | --- | | | | |
| **Interaction p-value:**1 | | | | | | | | | | | | | | | | | | | | | | | | | | | | | | | | | | | | | | |
| **IRAK-1 rs3027898 and miR-155 rs767649 cross-classification interaction** | | | | | | | | | | | | | | | | | | | | | | | | | | | | | | | | | | | | | | |
|  | | **AA** | | | | | | | | | | | **AT** | | | | | | | | | | | **TT** | | | | | | | | | | | | | | |
| **AA** | | 11 | 0 | | 1.00 | | | | | | | | 15 | 13 | | | | --- | | | | | | 0 | | | | | | 4 | | | | | --- | | | |
| **AC** | | 1 | 0 | | 1.51 | | | | | | | | 9 | 3 | | | | --- | | | | | | 0 | | | | | | 6 | | | | | --- | | | |
| **CC** | | 0 | 3 | | --- | | | | | | | | 4 | 3 | | | | --- | | | | | | 0 | | | | | | 0 | | | | | --- | | | |
| **Interaction p-value: 0.0067** | | | | | | | | | | | | | | | | | | | | | | | | | | | | | | | | | | | | | | |
|  | | | | | | | | | | | | | | | | | | | | | | | | | | | | | | | | | | | | | | |

The association analysis was performed using SNPStats online software. All interactions were performed in the codominant model (control, n=40 vs. MS, n=32). ^a^ adjusted for age in a logistic regression model. OR and *P*-values in bold are statistically significant, *P* < 0.05.

**
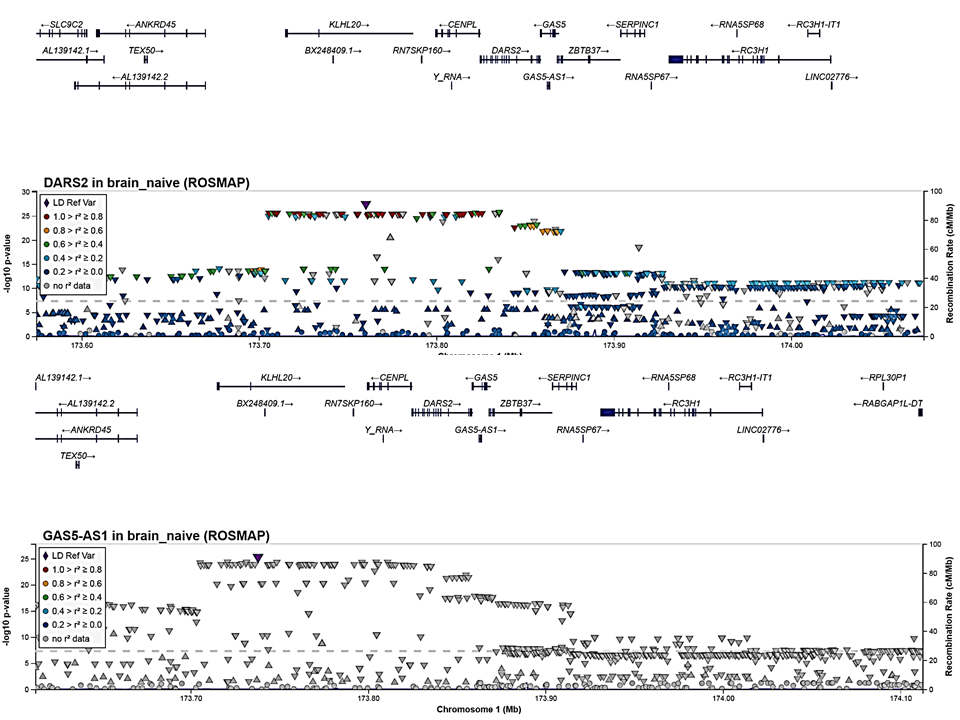
**

**Figure S1. Single-tissue eQTLs near *DRAS2* and *GAS5-AS1*.** A locus-centric view of eQTL *P*-values in the region around *DRAS2* and *GAS5-AS1* in the brain. Data are extracted FIVEx browser.

**
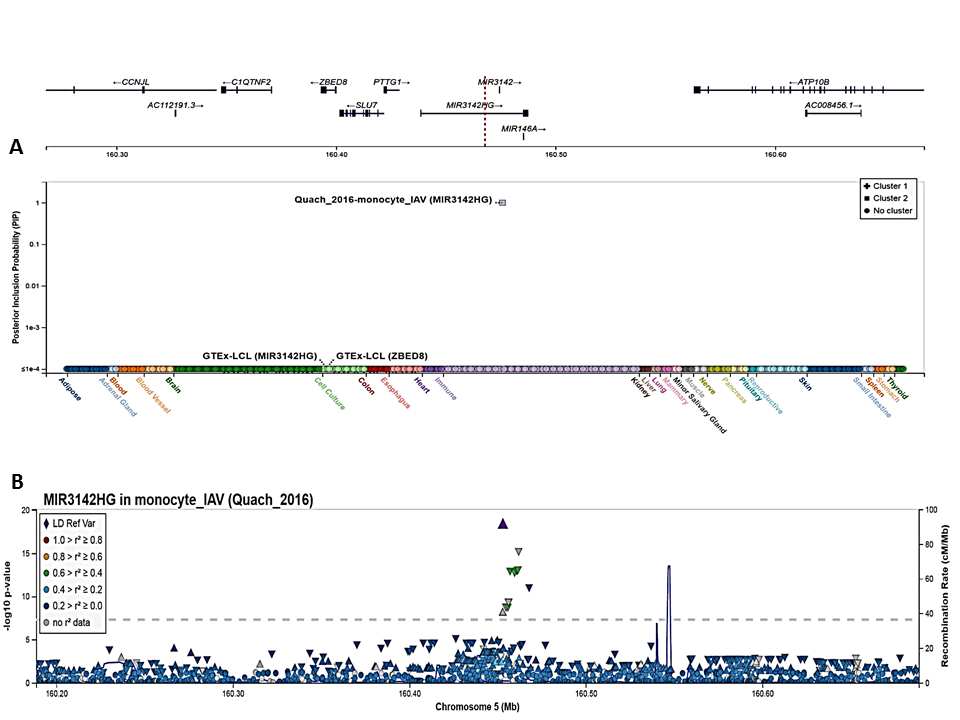
Figure S2. Single-tissue eQTLs near *MIR3142HG*, the host gene for *miR-146a*.** A) Posterior inclusion probability (PIP) to inform whether the variant is likely a causal variant. Indeed, PIP = 1 highlighting a strong signal in monocytes, indicating this variant (rs57095329) might be a causal variant. B) A locus-centric view of eQTL *P*-values in the region around MIR3142HG in monocytes. Data are extracted FIVEx browser.


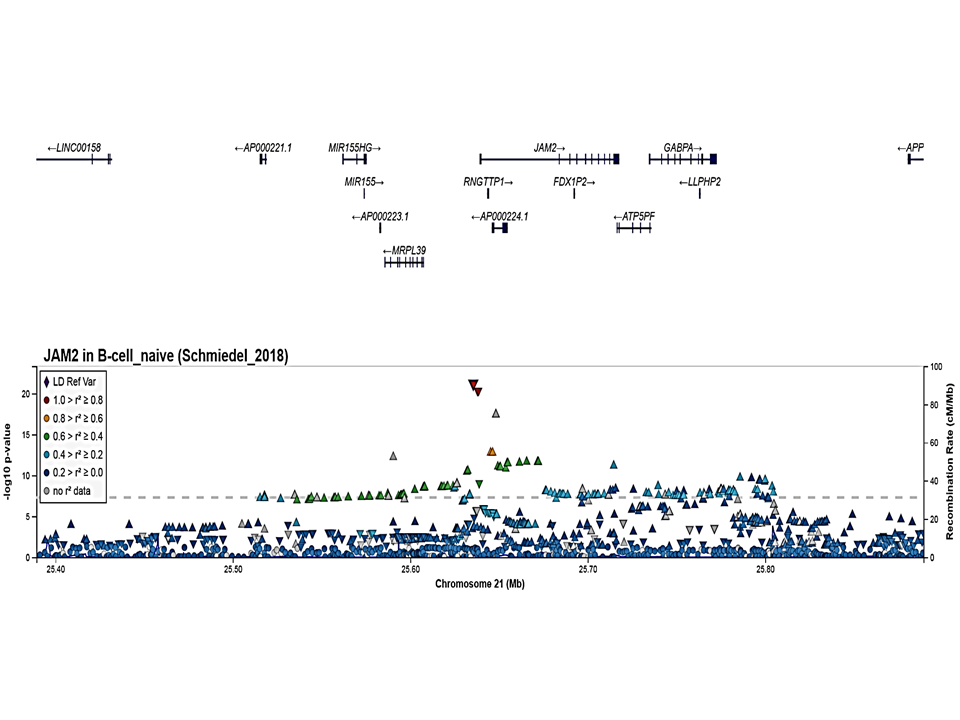


**Figure S3. Single-tissue eQTLs near *JAM2*.** A locus-centric view of eQTL *P*-values in the region around *JAM2* in B-cells. Data are extracted FIVEx browser.


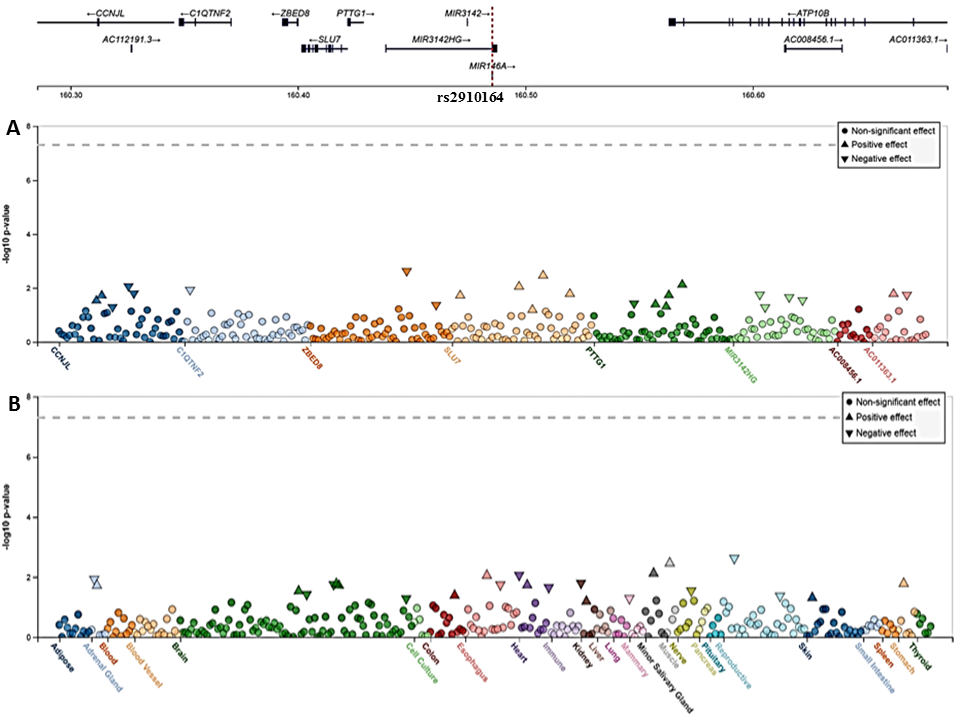


**Figure S4. cis-eQTLs associated with variant rs2910164 (5:160,485,411_C/G).** This variant does not show a strong eQTL for nearby genes in multiple systems. Data are extracted from FIVEx browser.


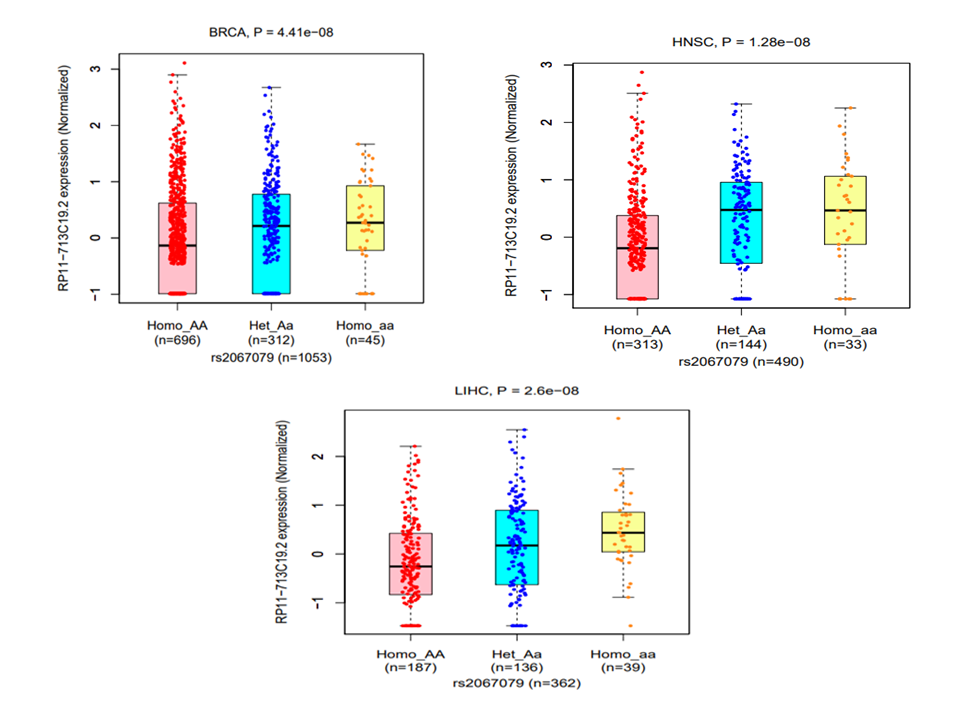


**Figure S5. Trans-eQTLs associated with variant rs2067079 (C/T).**. This SNP on chr1:173866073 has a significant trans-eQTL on *RP11-713C19.2* (ENSG00000213331, peroxiredoxin 6 pseudogene), expression (chr4:187970273~187971284:+) in breast cancer (BRCA), head and neck squamous cell carcinoma (HNSC), and liver hepatocellular carcinoma (LIHC). Homo_AA, the major homozygous genotype; Het_Aa, the heterozygous genotype; Homo_aa, the minor homozygous genotype. Data are extracted from the ncRNA-eQTL database.
